# Targeting protumor factor chitinase-3-like-1 secreted by Rab37 vesicles for cancer immunotherapy

**DOI:** 10.7150/thno.65522

**Published:** 2022-01-01

**Authors:** Pei-Shan Yang, Min-Hua Yu, Ya-Chin Hou, Chih-Peng Chang, Shao-Chieh Lin, I-Ying Kuo, Pei-Chia Su, Hung-Chi Cheng, Wu-Chou Su, Yan-Shen Shan, Yi-Ching Wang

**Affiliations:** 1 Department of Pharmacology, College of Medicine, National Cheng Kung University, Tainan, Taiwan.; 2 Institute of Clinical Medicine, College of Medicine, National Cheng Kung University, Tainan, Taiwan.; 3 Department of Clinical Medical Research, College of Medicine, National Cheng Kung University, Tainan, Taiwan.; 4 Department of Microbiology and Immunology, College of Medicine, National Cheng Kung University, Tainan, Taiwan.; 5 Institute of Basic Medical Sciences, College of Medicine, National Cheng Kung University, Tainan, Taiwan.; 6 Colorectal Division, Department of Surgery, National Cheng Kung University Hospital, College of Medicine, National Cheng Kung University, Tainan, Taiwan.; 7 Division of Oncology, Department of Internal Medicine, National Cheng Kung University Hospital, College of Medicine, National Cheng Kung University, Tainan, Taiwan.; 8 Division of General Surgery, Department of Surgery, National Cheng Kung University Hospital, College of Medicine, National Cheng Kung University, Tainan, Taiwan.

**Keywords:** chitinase 3-like-1, Rab37, exocytosis, neutralizing antibody, tumor microenvironment

## Abstract

**Background:** Chitinase 3-like-1 (CHI3L1) is a secretion glycoprotein associated with the immunosuppressive tumor microenvironment (TME). The secretory mode of CHI3L1 makes it a promising target for cancer treatment. We have previously reported that Rab37 small GTPase mediates secretion of IL-6 in macrophages to promote cancer progression, whereas the roles of Rab37 in the intracellular trafficking and exocytosis of CHI3L1 are unclear.

**Methods:** We examined the concentration of CHI3L1 in the culture medium of splenocytes and bone marrow derived macrophages (BMDMs) from wild-type or *Rab37* knockout mice, and macrophage or T cell lines expressing wild type, active GTP-bound or inactive GDP-bound Rab37. Vesicle isolation, total internal reflection fluorescence microscopy, and real-time confocal microscopy were conducted. We developed polyclonal neutralizing-CHI3L1 antibodies (nCHI3L1 Abs) to validate the therapeutic efficacy in orthotopic lung, pancreas and colon cancer allograft models. Multiplex fluorescence immunohistochemistry was performed to detect the protein level of Rab37 and CHI3L1, and localization of the tumor-infiltrating immune cells in allografts from mice or tumor specimens from cancer patients.

**Results:** We demonstrate a novel secretion mode of CHI3L1 mediated by the small GTPase Rab37 in T cells and macrophages. Rab37 mediated CHI3L1 intracellular vesicle trafficking and exocytosis in a GTP-dependent manner, which is abolished in the splenocytes and BMDMs from *Rab37* knockout mice and attenuated in macrophage or T cell lines expressing the inactive Rab37. The secreted CHI3L1 activated AKT, ß-catenin and NF-κB signal pathways in cancer cells and macrophages to foster a protumor TME characterized by activating M2 macrophages and increasing the population of regulatory T cells. Our developed nCHI3L1 Abs showed the dual properties of reducing tumor growth/metastases and eliciting an immunostimulatory TME in syngeneic orthotopic lung, pancreas and colon tumor models. Clinically, high plasma level or intratumoral expression of CHI3L1 correlated with poor survival in 161 lung cancer, 155 pancreatic cancer and 180 colon cancer patients.

**Conclusions:** These results provide the first evidence that Rab37 mediates CHI3L1 secretion in immune cells and highlight nCHI3L1 Abs that can simultaneously target both cancer cells and tumor microenvironment.

## Introduction

Chitinase 3-like-1 (CHI3L1, also named YKL-40 in humans and BRP-39 in mice) is a member of the glycosyl hydrolase 18 gene family and binds to chitin without degrading it 1. CHI3L1 is expressed and secreted by a variety of cells, including macrophages, T cells, neutrophils, epithelial cells, smooth muscle cells, fibroblasts and cancer cells 1-5. CHI3L1 exerts pleiotropic effects which may be mediated *via* interaction with several receptors to regulate wide range of functions in immune responses and extracellular matrix assembly in chronic inflammations, neurodegenerative diseases and cancer 4.

CHI3L1 is a protumor secretion protein and is associated with several signal pathways in the process of cancer development. The receptors of CHI3L1 include interleukin-13 receptor alpha 2 (IL-13Rα2), TMEM219, CD44 and galectin-3 5-7. CHI3L1 forms a multimeric complex with IL-13Rα2 and IL-13 to activate the MAPK/ERK, AKT, and Wnt/β-catenin cell signaling pathways to regulate growth and metastasis 6, 8. Furthermore, CHI3L1 and VEGF synergistically promote angiogenesis and malignancy in glioblastoma and melanoma 9-11. These reports suggest that CHI3L1 plays a critical role in tumorigenesis.

Creation of an immunosuppressive tumor microenvironment (TME) has been suggested as a possible mechanism of CHI3L1-induced cancer promotion. CHI3L1 mediates tumor progression and metastasis through the regulation of macrophage recruitment to reprogram to an M2-like phenotype 12, 13. For example, CHI3L1 binds with galectin-3, a protein highly expressed in M2 macrophages and extracellular milieu of glioblastoma TME, to regulate macrophage switch and pro-tumor immunity in glioblastoma 14. Additionally, CHI3L1 plays a significant role in CD4^+^ T cell development and T helper 2 (Th2)-mediated inflammation, while CHI3L1 decreases responses of Th1 and cytotoxic CD8^+^ T cells during cancer progression 2. Therefore, CHI3L1 drives immune cell activation and differentiation.

Elevated levels of CHI3L1 in the serum or plasma found in a number of solid tumors correlate with shorter overall survival 4, 15. In addition, CHI3L1-derived from cancer-associated fibroblasts or macrophages elicits an aggressive tumor phenotype such as tumor metastases and treatment resistance 12, 16. Although it is known that CHI3L1 is a protumor secretion factor, the mechanism of exocytosis of CHI3L1 remains undefined. In addition, the secretory mode of CHI3L1 makes it a promising target for cancer treatment as shown by several chemical inhibitors of CHI3L1 17, 18 and neutralizing antibodies 19, 20 developed.

Here, we report that Rab37 small GTPase, a regulator of vesicle trafficking and protein transportation 21, 22, mediates secretion of CHI3L1 in CD4^+^, CD8^+^ T cells and macrophages to foster an immunosuppressive TME. In this study, we also show that a mouse anti-human CHI3L1 polyclonal neutralizing antibody (nCHI3L1 Ab) largely attenuated tumor growth and metastasis *in vitro* and *in vivo* using three cancer models including lung, pancreatic and colorectal cancers. Importantly, we detect an increased plasma CHI3L1 concentration that correlates with poorer overall survival in our in-house cohort of non-small cell lung cancer (NSCLC), pancreatic ductal adenocarcinoma (PDAC) and colorectal cancer (CRC) patients. Our findings reveal the secretion mechanism of CHI3L1 and potential use of nCHI3L1 Ab for cancer treatment.

## Materials and Methods

### CD4^+^ and CD8^+^ T cells isolation and stimulation

*Rab37* knockout (KO) mice were generated as described in Kuo's report 23. CD4^+^ and CD8^+^ T cells were isolated from splenocytes derived from wild-type (WT) and *Rab37* KO mice using anti-mouse CD4 magnetic particles (BD Bioscience, #551539) and mouse CD8 T lymphocyte enrichment set (BD Bioscience, # 558471) according to the manufacturer's instructions. Cells were cultured in medium containing 10% Fetal Bovine Serum (FBS, Gibco), 1% penicillin/streptomycin (Gibco), and 1% sodium pyruvate (Gibco) and incubated at 37 ^o^C with 5% CO_2_ in air. Purified naïve CD4^+^ and CD8^+^ T cells were stimulated with 2 μg/mL anti-CD3/CD28 antibodies (BD Bioscience, #553057/ #553294) for 24 h or 48 h.

### Bone-marrow-derived macrophages (BMDMs) isolation and culture conditions

Bone marrow cells were aseptically harvested from hind legs of 6-8-wk-old WT or *Rab37* KO mice. All muscle tissues were removed and the femur and tibia were separated. Isolated bones were washed with DMEM containing 10% FBS (Gibco), 1% penicillin/streptomycin (Gibco) and 10 *n*g/mL macrophage colony-stimulating factor (M-CSF) (Peprotech, Somerset). Approximately 3 × 10^6^ isolated bone marrow cells were cultured in 10 cm dish. BMDMs were cultured for 7 days at 37 ^o^C with 5% CO_2_ in air and culture media was replaced with fresh media every 2 days before further experiments.

### Cytokine and chemokine array

Rab37 positive-related cargo proteins in supernatants of stimulated splenic T cells derived from WT and *Rab37* KO mice were detected with mouse cytokines and chemokines array-membrane (111 targets) (#ARY028, R&D systems). Supernatants derived from WT and *Rab37* KO stimulated splenic T cells (5 x 10^5^) were collected and filtered through sterile Millex^®^ Filter Unit (Merck Millipore, Cork, Ireland). The array was performed according to the manufacturer's instructions (Figure S1A-B).

### Measurement of mouse plasma cytokines by cytometric bead array

Peripheral blood samples were collected from mice to analyze the Th1/Th2/Th17 cytokines using the mouse Th1/Th2/Th17 set of cytometric bead array (BD Biosciences) according to manufacturer's instructions. In brief, seven groups of beads with distinct fluorescence intensities have been coated with capture antibodies specific for IL-2, IL-4, IL-6, IFN-γ, TNF-α, IL-17A, and IL-10 proteins. The concentration of each cytokine was determined by analyzing PE fluorescence intensity using CytoFLEX flow cytometer (Beckman Coulter).

### Enzyme-linked immunosorbent assay (ELISA)

The human CHI3L1 concentration in the conditioned medium (CM) from Jurkat cells and patient plasma was detected by a double antibody sandwich ELISA according to the manufacturer's instructions (DY2599, R&D systems). Identically, the mouse CHI3L1 concentration in CM from RAW264.7, BMDM or splenic T cells and mice plasma was detected (DY2649, R&D systems). The plasma samples were diluted to 1:50 before assay. The reaction was read at an OD of 450 nm using an ELISA plate reader. Each test included a standard control.

### Preparation of the mouse anti-human CHI3L1 polyclonal neutralizing antibodies (nCHI3L1 Abs)

Recombinant human CHI3L1 protein (rCHI3L1) was purchased from Leadgene Biomedical, Inc. (Tainan, Taiwan; #65013). To generate nCHI3L1 polyclonal antibodies, BABL/c mice were immunized twice with rCHI3L1 (1 mg/mice) at 4-6 week interval. Eight to ten weeks after immunization, the anti-sera of all mice were collected to purify the CHI3L1 specific antibodies *via* an antigen affinity column. The purity of antibodies was confirmed by SDS-PAGE. The binding specificity of these antibodies to CHI3L1 was determined by ELISA (Figure S2).

### Cell lines, plasmids, siRNAs, transfection and culture conditions

Human NSCLC cell lines A549 and H1299, human PDAC cell lines MIA PaCa-2 and PANC-1, mouse CRC cell line MC38, human Jurkat T cells and THP-1 monocytes, and mouse macrophage cell line RAW264.7, T cell line EL4, Lewis lung carcinoma (LLC) were purchased from the American Type Culture Collection. A549, H1299, LLC, MC38, and RAW264.7 were maintained in DMEM (Gibco) while MIA PaCa-2, PANC-1, Jurkat, EL4 and THP1 were in RPMI (Gibco) with 10% FBS (Gibco) and 1% penicillin/streptomycin (Gibco). Human umbilical vein endothelial cell line (HUVEC) was provided by Dr. Li-Wha Wu (Institute of Molecular Medicine, National Cheng Kung University, Taiwan). HUVECs cultured in dishes (coated with 0.1% gelatin for 1 h) were maintained with endothelial cell growth medium-2 (EBM-2; Lonza) and supplemented with SingleQuots^TM^ growth factor kit (Lonza). All cells were incubated at 37 °C with 5% CO_2_.

cDNA of human and mouse *Rab37* were purchased from OriGene and PCR fragments amplified with primers containing designated mutation for Rab37^Q89L^ and Rab37^T43N^ were cloned into the pcDNA-His-V5 vector (Invitrogen) or pDsRed2-C1 vector (NovoPro Bioscience Inc.). Human and mouse plasmid pCMV3-CHI3L1-HA and pCMV3-CHI3L1-GFPspark were purchased from Sino Biological Inc. Plasmid transfection was carried out with IT-J reagent (Mirus Bio) or lipofectamine (Invitrogen) The siRab37 were purchased from Dharmacon (siGENOME Human RAB37 siRNA, SMARTPool Cat. M-008933-02-0005). Plasmids used in these studies are listed in Table S1.

### Vesicle isolation and immunoprecipitation (IP)

Vesicle isolation protocol was as described in our previous study 23. Jurkat (2 × 10^6^), THP1 (1 × 10^7^) or RAW264.7 cells (1 × 10^7^) were sonicated and subjected for vesicle enrichment by two-speed of centrifugations (3,000 *g* for 10 min followed by 30,000 *g* for 60 min at 4 °C) using a 40-Ti rotor (Beckman). The vesicles-containing solution (500 μg) was incubated with anti-V5-tag antibody to isolate Rab37-specific vesicles and the CHI3L1 cargos in vesicles were analyzed by immunoblotting (Table S2).

### Confocal fluorescence microscopy and real-time imaging

Cells (1 × 10^4^) were fixed, incubated with primary and then secondary fluorescent antibodies (Table S2), and analyzed by Olympus FV3000 confocal microscope for the localization of Rab37 or CHI3L1. For real-time live confocal experiments, RFP-Rab37 and GFP-CHI3L1 were transfected into RAW264.7 cells 24 h prior to the experiment. The RFP-Rab37 and GFP-CHI3L1 signals were recorded through real-time live image and video through the 100× lense of the microscope.

### Total internal reflection fluorescence (TIRF) imaging

TIRF analysis was modified from Tsai's report 24. RAW264.7 macrophage cells were transfected with GFP-CHI3L1 and RFP-Rab37 for 16 h and 1 × 10^4^ cells were re-seeded in 3.5 cm glass bottom dish. TIRF microscopy system (Olympus IX81) equipped with a high sensitivity EMCCD Camera (iXOn3897, Andor technology) and a UPON 100X oil objective lens (NA = 1.49, Olympus) was used to capture 100-200 nm images below the plasma membrane. We defined each green fluorescence spot as a CHI3L1-containing vesicle, and then tracked each vesicle trafficking distance with trackIT software (Olympus).

### Transmission electron microscopy (TEM) imaging

For immuno-TEM analysis, splenocytes and BMDMs were fixed, sectioned, and immersed in H_2_O_2_ for 10 min, blocked with 1% BSA for 1 h and then incubated with indicated antibodies and protein A coupled to 10 nm or 20 nm gold particles (Table S2). Sections were post-stained with uranyl acetate and lead citrate and then examined under the transmission electron microscope JEM-1400 (JEOL, Japan).

### Immunofluorescence (IF), multiplex fluorescence immunohistochemistry (IF-IHC) and regular IHC

For IF staining, Opal stain kit (#NEL810001KT, Akoya Biosciences) was employed according to the manufacturer's instruction. The slides were stained with β-catenin or NF-κB primary antibody at 4 °C overnight. After Opal staining process, DAPI was applied for nuclei staining. Multiplex IF-IHC were conducted to examine the localization of Rab37, CHI3L1, CD8^+^ T cells, Tregs, F4/80, CD86^+^, CD206^+^ or CD163^+^ cells in allografts from mice or tumor specimens from cancer patients. Whole slides were scanned at 10× magnification (OLYMPUS cellSens) for visualization of the tumor, three non-overlapping regions of interest (ROIs) were selected and scanned at 20× for quantification. The size of the ROI was 200 x 200 µm (0.04 mm^2^) in allograft from mice, and 450 × 450 µm (0.2 mm^2^) in clinical patients' tissue slides. The detailed antibodies conditions are listed in Table S2.

### Cell proliferation, transwell migration, wound healing and tube formation assays

Cells proliferation assay was performed to evaluate the cell growth of nCHI3L1 Ab-treated groups by using cell counting kit 8 (CCK-8) assay (Dojindo, Kumamoto, Japan) on *ex vivo* isolated splenocytes and BMDMs as well as cultured cancer cells. Cancer cells were seeded in 96 well plates and cultured for 72 h followed by addition of 10 μL of the CCK-8 solution for incubation at 37 °C for 1 h. The reaction was read at an OD of 450 nm using an ELISA plate reader. Transwell migration and wound healing assays with cancer cells were performed as described 24. At the end of 16 h incubation, cells attached on the reverse side were fixed and then stained. For tube formation assay, phenol Red-free Matrigel (Corning) was added to 96-well plates and then incubated at 37 °C for 1 h. HUVEC cells (1 x 10^4^ per well) were seeded into 96-well plates with culture medium containing nCHI3L1 Ab or not and then incubated for 6 h. For HUVECs migration, HUVECs (2 x 10^5^) were placed in the upper chamber with serum-free EGM2 while the lower chamber was filled with lung cancer cells (2 x 10^5^) as chemoattractants and incubated with rCHI3L1 plus nCHI3L1 Ab or bevacizumab at 37 ^o^C for 16 h. The cells attached on the reverse side of the membrane were stained with crystal violet and counted under inverted microscope (Nikon E400, Tokyo, Japan). Three to six random selected views were photographed and quantified 25.

### Fluorescence-activated cell sorting (FACS) analysis

For binding ability of CHI3L1 to receptor, NSCLC cell line H1299 was harvested and thoroughly mixed with His-tagged rCHI3L1 or nCHI3L1 Ab for 1 h. Subsequently, cells were incubated with FITC-labeled 6x-His Tag antibody at 4 °C for 1 h and analyzed by FACS. To determine PD-1 and TIM-3 presentation on CD8^+^ T cells, cells were stained using anti-human PD-1-BB515 (BD Bioscience) and TIM-3-BV421 (BD Bioscience) antibodies. After cell surface staining, intracellular CD107a was stained with CD107a-PE (BD Bioscience) antibodies to detect T cell activities.

Allograft tumor tissues were digested with 0.1 mg/mL collagenase (Sigma-Aldrich) and 1 mg/ml dispase II (Sigma-Aldrich) at 37 ℃ for 30 min and meshed. Resulting single-cell suspension were stained with CD4, CD8, CD11b, CD25, CD86, CD206, CTLA4 and Foxp3 antibody (BD Bioscience). The data were recorded by CytoFLEX (Beckman Coulter). The detailed antibodies conditions are listed in Table S2.

### Allograft tumor growth and metastasis assays* in vivo*

All animal experiments were performed in compliance with National Cheng Kung University (NCKU) institutional guidelines for use and care of animals (Permit Numbers: #106255). All mice used in the experiments were 6-wk-old male C57BL/6 mice. For lung tumor subcutaneous model (*n =* 10 mice per group), 5 × 10^5^ LLC cells were subcutaneously injected into flanks of mice. For lung orthotopic model (*n =* 5 mice per group), 1 × 10^4^ LLC cells in 100 μL PBS were implanted into the left lobe of the lung in mice. For pancreas and colorectal orthotopic tumor models, intra-pancreas and intra-cecum injection of 5 × 10^6^ KPPC-Luc (*n =* 6 mice per group for anti-tumor assay; *n =* 10 for survival analyses) and 1 × 10^6^ MC38 cells (*n =* 5 mice per group) were performed on mice, respectively. For intravenous (i.v.) injection CRC models, 1 × 10^6^ MC38 cells (*n =* 5 mice per group) in 100 μL PBS were i.v. injected *via* the tail vein into mice. In all models, 5 μg per gram mouse body weight of IgG isotype antibody or nCHI3L1 antibody were given by i.v. injection* via* the tail vein into mice harboring allograft. Antibodies were given by i.v. at day 3 after cancer cell inoculation and once every 3 days for a total of four to five times. For subcutaneous model, tumor volume was calculated at day 6 and once every three days using the equation V = (a × b × c) during observation. For orthotopic model, mice were sacrificed on day 20 (NSCLC), day 28 (PDAC), or day 27 to 33 (CRC) and the primary tumor and potential metastatic organs were removed and resected for protein extraction, FACS analysis and preserved in paraffin for IHC or IF-IHC staining. For toxicity tests, blood samples and major organs were collected for serum biochemistry and histology examinations, respectively.

### Patient samples and clinical information

Surgically resected NSCLC patients (*n =* 161), PDAC patients (*n =* 155), and CRC patients (*n =* 180) were recruited from NCKU Hospital, after obtaining appropriate institutional review board permission (#B-ER-106-102) and informed consent from the patients. Overall survival was calculated from the day of surgery to the date of death or the last follow-up. The end of the follow-up was defined as February 2021. A total of 11 lung cancer patients receiving clinical α-PD-1 Ab treatment were also recruited. All patients received regular follow-up at 3-month interval to evaluate treatment responses. The detailed clinicophathological characteristics of the enrolled patients are listed in Table S3.

### Statistics

Cell studies were conducted in three independent experiments unless indicated otherwise. The number of mouse or patient per group for animal and clinical studies was described for each experiment. The one-way ANOVA test was used for multiple comparisons. Two-tailed Student's *t* test was used when one comparison was being made. Data represented mean ± SD. Pearson χ^2^ test was used to compare the correlation of CHI3L1 with clinicopathological parameters. Correlation between Rab37, CHI3L1 or CD163 protein expression was calculated by SPSS scatter plot and Pearson correlation assay. Overall survival curves were calculated according to the Kaplan-Meier method, and comparison was performed using the log-rank test. Cox regression comparison was performed to analyze the relative risk for patient poor outcome. The level of statistical significance was taken as *p* value, *, *p* < 0.05; **, *p* < 0.01; ***, *p* < 0.001.

## Results

### Cytokine/chemokine array and ELISA identify CHI3L1 as a cargo of Rab37 in T cells and macrophages

CHI3L1 secreted glycoprotein is known to play a significant role in the pathogenesis of Type 2-mediated inflammation and cancer; however, its trafficking mode remains undefined. We first attempted to investigate the secretion mechanism of CHI3L1. Several exocytic Rab small GTPases have been reported to target the release of immune modulating proteins to shape the TME 26. We previously reported that Rab37 acts as a tumor promoter in macrophages by mediating IL-6 secretion to activate M2 macrophage polarization and reprogram PD-1^+^CD8^+^ exhausted T cells, ultimately fostering an immunosuppressive TME 23. We therefore tested whether Rab37 mediates the exocytosis of protumor secretion factor CHI3L1. To identify the potential cargos of Rab37, conditioned media (CM) from anti-CD3/CD28 antibodies stimulated splenic T cells derived from wild-type (WT) and *Rab37* knockout (KO) mice were collected and subjected to cytokine/chemokine array analyses (Figure S1A-B). CHI3L1 was one of the top proteins decreased in CM from *Rab37-*KO splenic T cells. CHI3L1 is known as a protumor secretion factor, however, the mechanism of exocytosis of CHI3L1 remains undefined.

**Figure 1:**
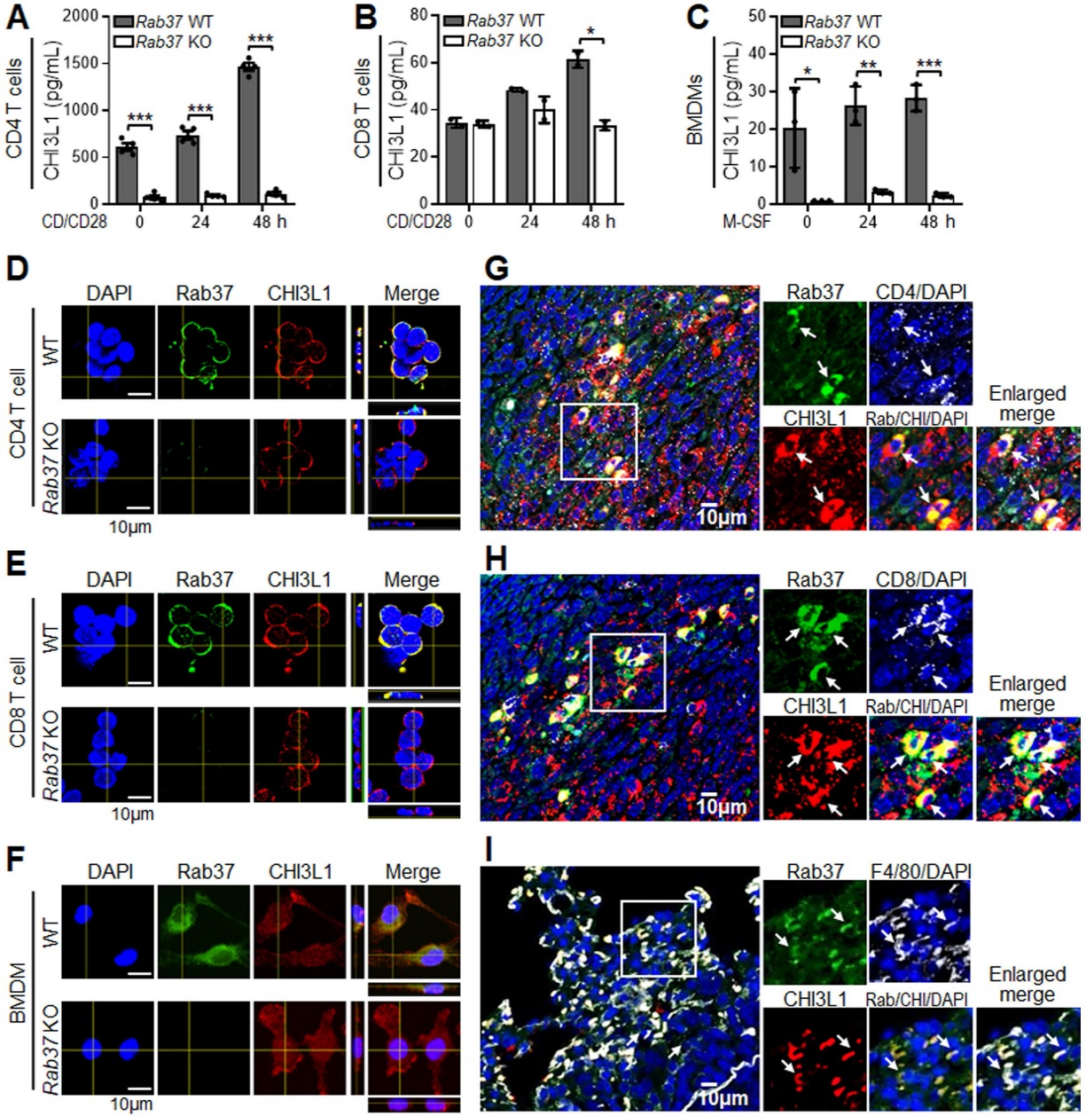
** Rab37 colocalizes with CHI3L1 in macrophages and T cells.** (**A-C**) ELISA was performed to validate the level of CHI3L1 in CM from anti-CD3/CD28 antibodies stimulated CD4^+^ T cells (A), CD8^+^ T cells (B) or M-CSF differentiated BMDMs (C) isolated from *Rab37* WT and KO mice* ex vivo*. (**D-F**) Confocal microscopy images of intracellular Rab37 (green) and CHI3L1 (red) and nuclear staining (blue) in* Rab37* WT and KO CD4^+^ T cells (D), CD8^+^ T cells (E) or BMDMs (F). Z-stack images are shown. Scale bars: 10 μm. (**G-I**) Colocalization of Rab37 and CHI3L1 in CD4^+^ T cells (G), CD8^+^ T cells (H) and F4/80^+^ macrophage (I) by immunofluorescent-immunohistochemistry (IF-IHC) in orthotopic LLC lung tumor model. Enlarged images of the inset of merged panel are shown. Arrow indicates colocalization. Scale bars: 10 μm. Data represent mean ± SD. * *p* < 0.05; ** p < 0.01; *** p < 0.001, Student's *t*-test.

To further assess whether CHI3L1 is secreted by Rab37, ELISA assay was performed. The secretion levels of CHI3L1 were decreased in CD4^+^ T cells (**Figure 1A**), CD8^+^ T cells (**Figure 1B**) and BMDMs (**Figure 1C**) from *Rab37* KO mice compared to WT mice. Immunofluorescence (IF) confocal images demonstrated that Rab37 co-localized with CHI3L1 in CD4^+^ T cells, CD8^+^ T cells and BMDMs derived from WT mice (*Upper*, **Figure 1D-F**), while no co-localization signal was observed in *Rab37* KO-derived cells (*Lower*, **Figure 1D-F**). Importantly, the relationship between Rab37 and CHI3L1 expression has never been examined in infiltrating immune cells in tumor tissue. Therefore, we performed multiplex immunofluorescence immunohistochemistry (IF-IHC) on Lewis lung carcinoma (LLC) orthotopic allografts taken from *Rab37* WT mice. The results demonstrated that high Rab37 expression showed concordantly increased CHI3L1 level in the tumor infiltrated CD4^+^ T cells (**Figure 1G**), CD8^+^ T cells (**Figure 1H**) and F4/80^+^ macrophages (**Figure 1I**). These *in vivo* results corroborated with the *in vitro* observations that Rab37 colocalized with CHI3L1 in immune cells in TME.

### Rab37 mediates CHI3L1 secretion in a GTPase dependent manner

**Figure 2:**
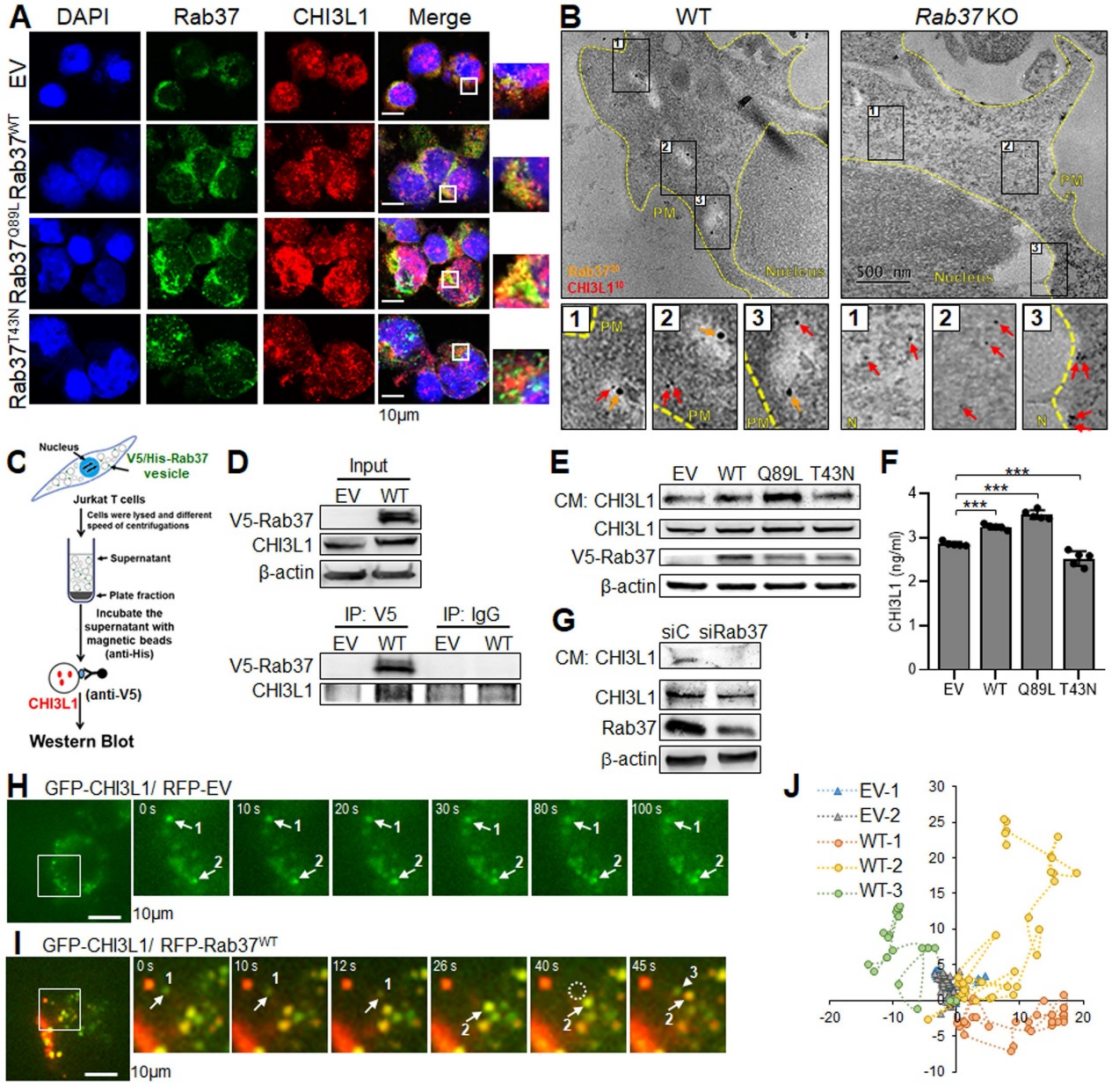
** Rab37 mediates CHI3L1 trafficking in T cells in a GTPase-dependent manner.** (**A**) Confocal microscopy images of Rab37 (green), CHI3L1 (red) and nuclear staining (blue) in EV, Rab37^WT^, Rab37^Q89L^ or Rab37^T43N^ Jurkat T cells. Enlarged images of merged panel are shown. Scale bars: 10 μm. (**B**) Ultrastructural localization of Rab37 (20 nm of gold, yellow arrow) and CHI3L1 (10 nm of gold, red arrow) illustrated by immuno-EM images of control *Rab37* WT (*Left*) or KO (*Right*) splenocytes (H). Scale bars: 500 nm. Enlarged images shown in insets of three representative regions. (**C**, **D**) Vesicles of Jurkat cells expressing V5-tagged Rab37 or EV were collected by centrifugations and immunoprecipitated (IP) with anti-V5 and vesicle lysates were blotted for V5-Rab37 and endogenous CHI3L1. (**E**, **F**) CM-WB (E) and CM-ELISA (F) were performed to validate the level of CHI3L1 in CM from EV, Rab37^WT^, Rab37^Q89L^ or Rab37^T43N^ Jurkat T cells. (**G**) CM-WB of CHI3L1 in si-Rab37 Jurkat T cells. (**H**-**J**) Selected frames from TIRF movies of GFP-tagged CHI3L1 co-transfected with RFP-tagged RFP-EV (H) or Rab37^WT^ (I) RAW264.7 cells. Enlarged images of the boxed areas with time intervals in seconds are shown (*Right*). Arrow indicates trafficking vesicle. Scale bars: 10 μm. (J) For quantification of vesicle trafficking event, we defined two vesicles for EV group (EV-1 and EV-2) and three vesicles for Rab37^WT^ (WT-1, WT-2 and WT-3), and then tracked each particle trafficking distance with trackIT software. Data represent mean ± SD. * *p* < 0.05; ** p < 0.01; *** p < 0.001, one-way ANOVA test.

These results prompted us to determine the trafficking mechanism of Rab37-mediated CHI3L1 secretion. Since Rab37 is a small GTPase regulated by GTP or GDP, we expressed empty vector control (EV), Rab37 wild-type (Rab37^WT^), Rab37-GTP constitutively active mutant (Rab37^Q89L^) and Rab37-GDP dominant negative mutant (Rab37^T43N^) in human Jurkat T cell line or murine RAW264.7 macrophage cell line. The merge panel of IF images illustrated that Rab37 co-localized with CHI3L1 to form yellow fluorescent puncta in Jurkat T cells expressing Rab37^WT^ and Rab37^Q89L^ constitutively active mutant, whereas a few co-localized vesicles formed in cells expressing EV and Rab37^T43N^ (**Figure 2A**). We observed similar colocalization results in RAW264.7 macrophage cells (Figure S3A). To further confirm the ultrastructure of CHI3L1 colocalization in Rab37-mediated vesicle, we performed immuno-TEM of Rab37 and CHI3L1 proteins in splenocytes or BMDMs from *Rab37* KO and WT mice. Strikingly, CHI3L1 was localized in Rab37-containing vesicles in splenocytes or BMDMs from WT mice, while there was little, if any, colocalization of CHI3L1 with Rab37 in *Rab37* KO splenocytes or BMDMs (**Figure 2B** and Figure S3B). These imaging results demonstrate that CHI3L1 localizes in Rab37-associated vesicles in T cells and macrophages.

Next, we performed series biochemical analyses to investigate the functional role of Rab37-regulated CHI3L1 secretion in T cells and macrophages. Vesicle isolation analyses were conducted to confirm that CHI3L1 was a cargo protein presenting in the Rab37-containing vesicles. We first transfected V5-tagged Rab37 to Jurkat T cells and then isolated Rab37-specific vesicles using V5-tagged antibody to immunoprecipitate (IP) Rab37-specific vesicles followed by Western blotting to detect CHI3L1 protein level (**Figure 2C**). The results revealed that the protein level of CHI3L1 in Rab37-specific vesicles was elevated in Rab37^WT^ Jurkat cells compared to EV control cells (**Figure 2D**). In addition, CHI3L1 was also found in Rab37-coated vesicles in macrophage cell lines THP-1 and RAW264.7 (Figure S3C-D). These results confirmed that CHI3L1 localizes in Rab37-specific vesicles in T cells and macrophages.

To further determine whether Rab37 mediates CHI3L1 secretion in a GTPase-dependent manner, CM-immunoblot and CM-ELISA were performed. The CM derived from Jurkat T cells expressing EV, Rab37^WT^, Rab37^Q89L^ and Rab37^T43N^ were collected for CM-immunoblotting. The data showed that CHI3L1 secretion level was increased in CM from Rab37^WT^ or Rab37^Q89L^ constitutively active overexpressing cells compared to EV or Rab37^T43N^ dominant negative cells (**Figure 2E**). It is interesting to note that cytosolic CHI3L1 level remained steady in EV, Rab37^WT^, Rab37^Q89L^ and Rab37^T43N^ Jurkat T cells, suggesting that Rab37 regulates CHI3L1 secretion but not its level of protein expression. Moreover, CM-ELISA results showed that CHI3L1 secretion level was elevated in CM from Rab37^WT^ and Rab37^Q89L^, but decreased in Jurkat T cells Rab37^T43N^ (**Figure 2F**). Similar results were observed in THP-1 and RAW264.7 macrophage cell lines expressing EV, Rab37^WT^, Rab37^Q89L^ and Rab37^T43N^ (Figure S3E-F). On the contrary, CM-immunoblotting results showed a significant reduction of CHI3L1 secretion in Jurkat T cells knocking down of Rab37 (**Figure 2G**). Collectively, these results suggested that Rab37 regulates CHI3L1 exocytosis in T cells and macrophages in a GTPase-dependent manner.

### CHI3L1 is a trafficking cargo of Rab37 in splenocytes and macrophages

To visualize the dynamic trafficking of CHI3L1 by Rab37, we employed total internal reflection fluorescence (TIRF) microscopy to observe fluorescent-labelled signals in the proximity of the plasma membrane (PM). The RAW264.7 macrophage cells were co-transfected with GFP-tagged CHI3L1 (GFP-CHI3L1) and RFP-tagged Rab37 (RFP-Rab37). The TIRF images showed that colocalized signals (yellow) rapidly appeared and shifted in both horizontal and vertical directions in Rab37-WT cells (**Figure 2H**, **J**), whereas the fluorescence signals in cells co-transfected with RFP-EV control and GFP-CHI3L1 vectors barely moved (**Figure 2I**, **J**).

We also conducted time-lapse confocal microscopic analysis in RFP-EV or RFP-Rab37^WT^ RAW macrophage cells co-transfected with GFP-CHI3L1 (Figure S3G-H). In Rab37^WT^ RAW264.7 cells, CHI3L1 was rapidly colocalized with Rab37 puncta to form yellow vesicles. In EV RAW cells, RFP-EV (red) and GFP-CHI3L1 (green) signals appeared in distinct cellular compartment. Together, our results provided first evidence of trafficking dynamic of Rab37-mediated CHI3L1 intracellular transportation and exocytosis to the extracellular compartment.

### nCHI3L1 Abs increase anti-cancer immune cell response *ex vivo* and reduce NF-κB activity in macrophages

**Figure 3:**
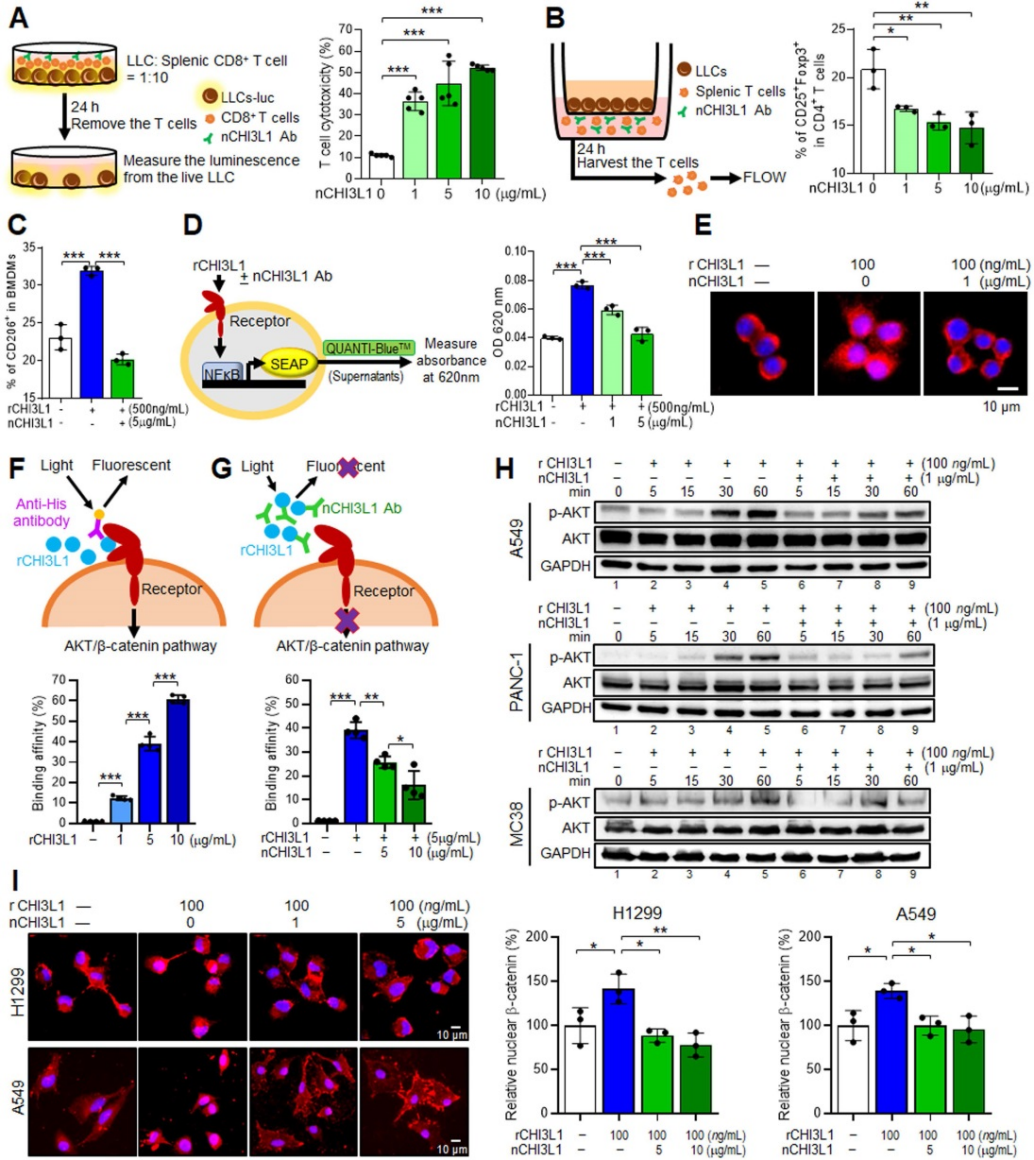
** nCHI3L1 Ab stimulates macrophage and lymphocyte immunity *ex vivo* and inactivates AKT, β-catenin and NF-κB *in vitro.*** (**A**) Luciferase-LLC cells (LLC-luc) were mixed and cultured with splenic CD8^+^ T cells at 1:10 ratios, and then treated with CHI3L1 neutralizing antibody (nCHI3L1 Ab) for 24 h (*Left*). Cancer cell viability was measured by luciferase assay (*Right*). (**B**) Scheme for *ex vivo* Treg differentiation assay by co-culturing splenic T cells with LLC (*Left*). nCHI3L1 Ab inhibited Treg differentiation measured by FACS assay (*Right*). (**C**) nCHI3L1 Ab inhibited recombinant CHI3L1 protein (rCHI3L1)-induced M2 (CD206^+^) polarization. (**D**) Scheme for NF-κB activity in RAW-Blue™ cells. Absorbance at 620 nm indicated the NF-κB activity (*Left*). nCHI3L1 Ab inhibited rCHI3L1-triggered NF-κB activation (*Right*). (**E**) IF staining of NF-κB (red) and nucleus (blue) in RAW264.7 macrophages after rCHI3L1, or rCHI3L1 and nCHI3L1 Ab treatment for 15 min. Scale bars: 10 μm. (**F, G**) The scheme depicting rCHI3L1 added to culture media of H1299 lung cancer cells emit fluorescence when bound to receptor in a dose dependent manner (F). nCHI3L1 Ab attenuated fluorescence, indicating that nCHI3L1 Ab blocked the interaction between rCHI3L1 and receptor (G). (**H**) nCHI3L1 Ab inhibited AKT phosphorylation induced by rCHI3L1 during the treatment period as indicated in A549 lung cancer, PANC-1 pancreatic cancer and MC38 colon cancer cells. (**I**) IF staining of β-catenin (red) and nucleus (blue) in H1299 and A549 lung cancer cells after rCHI3L1 and or nCHI3L1 Ab treatment for 1 h. Scale bars: 10 μm. Data represent mean ± SD. * *p* < 0.05; ** p < 0.01; *** p < 0.001, one-way ANOVA test.

CHI3L1 has been shown to mediate a number of pro-inflammatory effects as its secretion induces Th2 polarization and macrophage activation 2, 27. In addition, our cytokine/chemokine array results in *Rab37* KO mice model showed a positive correlation between decreased CHI3L1 level and Th1 inflammation program (Figure S1C). To impair the anti-inflammatory effects of CHI3L1, we developed polyclonal CHI3L1-neutralizing antibodies (nCHI3L1 Abs). We next sought to determine whether functions of T cells and macrophages can be reprogrammed by nCHI3L1 Abs. First, we investigated lymphocyte-mediated cytotoxicity against cancer cells by co-culturing luciferase-expressing LLC (LLC-luc) cells with CD8^+^ T cells isolated *ex vivo* (*Left,*** Figure 3A**). We observed that the lymphocyte-mediated cytotoxicity of LLC-luc was further promoted by treatment with nCHI3L1 Abs at a dose-dependent manner (*Right,*** Figure 3A**). Importantly, an increase in CD107a on CD8^+^ T cells upon nCHI3L1 Ab treatment indicated an enhanced CD8^+^ T cell activity (Figure S4A). In addition, large decrease in exhausted CD8^+^ T cell subpopulation (PD-1^+^Tim-3^+^CD8^+^) was observed in T cells treated with nCHI3L1 Abs (Figure S4B). Moreover, we determined the effect of nCHI3L1 Abs on differentiation of regulatory T (Treg) cells. We first used LLC co-culture to induce CD4^+^CD25^+^Foxp3^+^ Treg differentiation in *ex vivo* isolated splenic T cells (*Left,* **Figure 3B**). FACS analysis showed that nCHI3L1 Ab treatment decreased the percentage of Tregs (*Right,* **Figure 3B**). Notably, nCHI3L1 Ab treatment did not show cytotoxicity on splenic lymphocytes (Figure S4C). Together, these results revealed that nCHI3L1 Ab treatment enhanced CD8^+^ T cell activity and reduced Treg differentiation.

CHI3L1 regulates macrophage polarization to an M2-like phenotype 12, 13, we thus verified whether recombinant CHI3L1 proteins (rCHI3L1)-induced macrophages polarization could be reprogrammed by nCHI3L1 Abs. *Ex vivo* isolated BMDMs were treated with rCHI3L1 in the presence or absence of nCHI3L1 Abs. FACS analysis showed that the percentage of M2-like macrophages among the rCHI3L1-treated BMDMs was decreased upon nCHI3L1 Ab treatment (**Figure 3C**). Moreover, the RT-qPCR results demonstrated that nCHI3L1 Abs were able to reduce the expression of M2 marker gene *Arginase-1* (*Arg1*) in BMDMs, while did not show cytotoxicity in BMDMs (Figure S4D-E). NF-κB plays a complex role in the process of inflammation and development of cancer. Inhibition of NF-κB signaling in M2-polarized macrophages mediates pro-inflammatory responses upon exposure to pathogens 28. On the other hand, loss of NF-κB function reprograms tumor associated macrophages (TAMs) into M1-phenotype, suggesting a promoting role of NF-κB in maintaining the phenotype of M2-TAMs 29, 30. Notably, some studies suggest a positive feedback loop between CHI3L1 and the NF-κB signaling 17, 31. We next examined the relationship between CHI3L1 and NF-κB activity in macrophages. We incubated rCHI3L1 proteins with or without nCHI3L1 Abs in RAW-Blue™ cells, which is a macrophage cell line harboring a secreted embryonic alkaline phosphatase (SEAP) reporter construct inducible by NF-κB. We measured absorbance at 620 nm as the production of SEAP representing the NF-κB activity (*Left,* **Figure 3D**). Our results showed that nCHI3L1 Abs reduced binding of rCHI3L1 to RAW-Blue™ cells and subsequently decreasing the NF-κB activity (*Right,* **Figure 3D**). To further verify the effects of nCHI3L1 Ab treatment on NF-κB activity, RAW264.7 macrophage cells were treated with nCHI3L1 Abs while stimulated with rCHI3L1. IF staining results demonstrated that NF-κB translocated into the nucleus after addition of rCHI3L1, while NF-κB nuclear localization was attenuated upon nCHI3L1 Ab treatment (**Figure 3E**). Together, these results suggested that nCHI3L1 Ab treatment enhanced CD8^+^ T cell cytotoxicity and attenuated Treg and M2-type macrophage polarization to induce anti-tumor properties *in vitro*.

### CHI3L1 nAbs block the binding ability of recombinant CHI3L1 to inhibit AKT and β-catenin signals in various cancer cell lines

To impair the protumor effects of CHI3L1, we further examined the function of nCHI3L1 Abs on cancer models including NSCLC cells H1299 and A549, PDAC cells PANC-1 and MIA PaCa-2 as well as CRC cells MC38. First, we investigated whether these nCHI3L1 Abs blocked the binding of CHI3L1 to its receptor on H1299 cells by FACS analyses. We used anti-His antibody to recognize the His-tagged recombinant CHI3L1 (rCHI3L1) protein. We used anti-His antibody to recognize the His-tagged rCHI3L1 protein. CHI3L1 binding activity was increased in a dose-dependent manner on H1299 cancer cells (**Figure 3F** and Figure S4F), while nCHI3L1 Abs decreased the fluorescence signals induced by rCHI3L1 protein (**Figure 3G** and Figure S4G), suggesting that the nCHI3L1 Abs prevented CHI3L1 protein from binding to its receptor on H1299 cells.

CHI3L1 activates AKT signaling and induces β-catenin nuclear localization in mice and humans 8, 32. Thus, we tested whether nCHI3L1 Abs could inhibit AKT activation. The immunoblotting analyses indicated that nCHI3L1 Abs inhibited AKT phosphorylation induced by rCHI3L1 in various NSCLC, PDAC and CRC cells (*Lanes 6-9 vs 2-5*, **Figure 3H**, and Figure S4H), confirming the AKT inhibition function of nCHI3L1 Abs. Additionally, IF images showed that the nuclear localization of β-catenin was enhanced in H1299 and A549 cells treated with rCHI3L1, whereas decreased nuclear localization of β-catenin was observed in nCHI3L1 Abs-treated cells (**Figure 3I**). These results supported the anti-cancer signaling effects of nCHI3L1 Abs.

### nCHI3L1 Abs reduce cell growth and migration of cancer cells and endothelial cells *in vitro*

**Figure 4:**
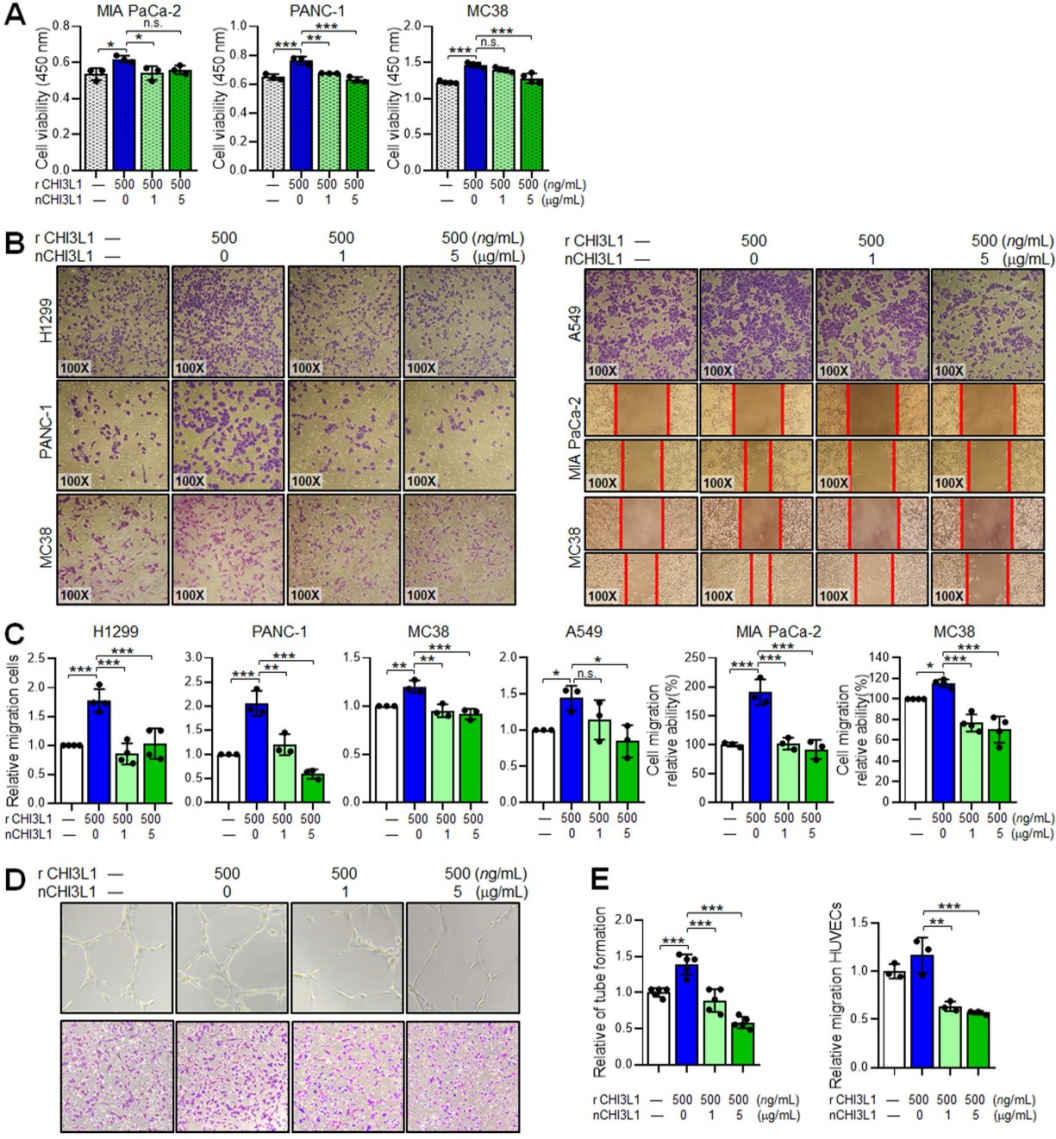
** nCHI3L1 Ab inhibits cell growth and migration in various cancer cell lines and reduces angiogenesis of endothelial cells *in vitro*.** (**A**) Cell viability assay showing nCHI3L1 Ab decreased the proliferation induced by rCHI3L1. (**B, C**) Transwell migration assay showing nCHI3L1 Ab reduced the migration ability triggered by rCHI3L1. (**D, E**) Tube formation (*Upper*) and transwell migration (*Lower*) assays (D) and quantitative results (E) showing nCHI3L1 Ab reduced the *in vitro* angiogenesis ability of HUVEC endothelial cells triggered by rCHI3L1. Data represent mean ± SD. * *p* < 0.05; ** p < 0.01; *** p < 0.001, one-way ANOVA test.

To determine the anti-cancer functions of nCHI3L1 Abs in cancer cells *in vitro*, PDAC cells MIA PaCa-2 and PANC-1 as well as CRC cells MC38 were treated with various doses of nCHI3L1 Abs for 72 h and then subjected to CCK-8 cell viability assay. Both doses of 1 and 5 μg/mL nCHI3L1 Ab treatments inhibited the viability of various cancer cell lines (**Figure 4A**). Further, we discovered that CHI3L1 nAb treatments (1 and 5 μg/mL) significantly decreased the migration ability of human NSCLC cell lines H1299 and A549 and PDAC cell lines MIA PaCa-2 and PANC-1 cells as well as CRC cell line MC38 induced by rCHI3L1 using transwell migration assay or wound healing assay (**Figure 4B-C**).

CHI3L1 has been shown to stimulate angiogenic responses in endothelial cells 9-11. We thus performed tube formation and transwell migration assays on HUVECs incubated with rCHI3L1 plus nCHI3L1 Abs or bevacizumab, clinical used VEGF-targeted therapeutic antibody 33. *In vitro* HUVEC tube formation and transwell migration assays showed that rCHI3L1 promoted HUVECs tube formation and migration, whereas co-incubation of nCHI3L1 Abs reduced HUVECs tube formation and migration in a dose-dependent manner compared with control group (**Figure 4D-E**). Treatment with bevacizumab served as positive control (Figure S5). These results confirmed that our in-house generated nCHI3L1 Abs reduced cancer cell growth and migration, and exerted anti-angiogenic responses in endothelial cells *in vitro*.

### nCHI3L1 Abs suppress tumor growth and metastasis in subcutaneous and orthotopic lung tumor models *in vivo*

Our *in vitro* and *ex vivo* data so far suggested that targeting CHI3L1 by nAbs inhibited cancer cell growth and migration and promoted immunostimulatory functions of T cells and macrophages. These results prompted us to determine whether CHI3L1 nAbs ameliorate the immunosuppressive environment *in vivo* in addition to their anti-tumor growth and metastasis effects in three preclinical models.

**Figure 5:**
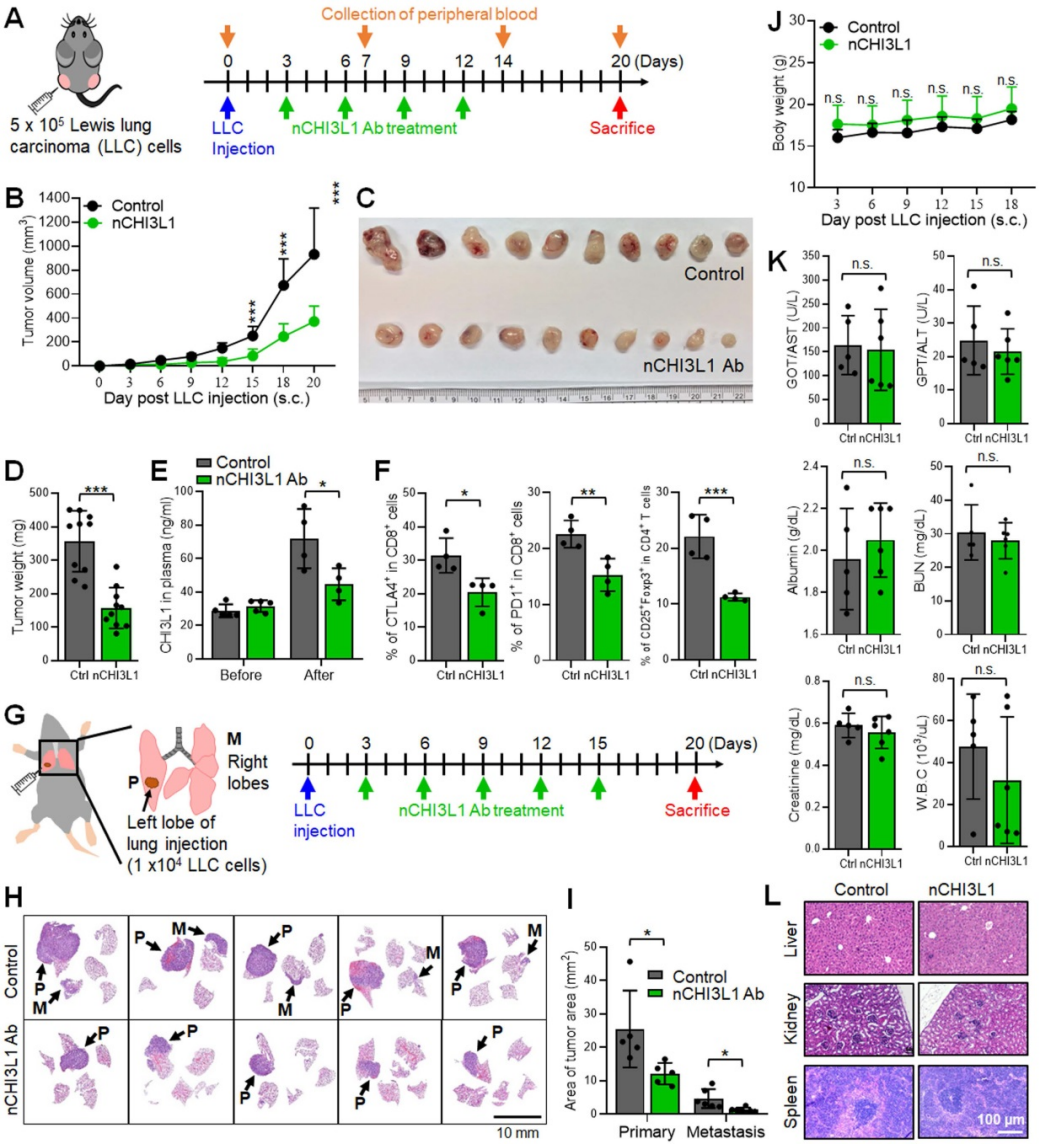
** nCHI3L1 Ab suppresses tumor growth and metastasis in subcutaneous and orthotopic lung tumor models *in vivo.* (A)** The scheme showing the LLC subcutaneous model in *C57BL/6* mice with nCHI3L1 Ab treatments. **(B-D)** Tumor volume (B), tumor size (C) and tumor weight (D) of LLC allograft in upon IgG (gray) or nCHI3L1 Ab (green) treatment. **(E)** Elevated CHI3L1 plasma level was observed in the control group, while maintained at the normal range from nCHI3L1 Ab-treated mice. (**F**) Tumor-infiltrating CTLA-4^+^CD8^+^ cells (*Left*) or PD-1^+^CD8^+^ cells (*Middle*) and CD4^+^CD25^+^Foxp3^+^ Tregs (*Right*). **(G)** The scheme showing the lung orthotopic model with LLC injected into the left lung. **(H)** Representative H&E-stained lung sections of primary tumor (P) in the left lung and metastatic tumors (M) in the right lateral lungs in mouse of IgG (*Upper*) or nCHI3L1 Ab (*Lower*) treatment group. Scale bar: 10* n*m. **(I)** Overall area of tumor nodules of primary tumors and metastatic tumors. **(J-L)** The body weight (J), serum biochemical markers (K) and major organs (L) of treated mice examinations revealed no significant adverse effects upon nCHI3L1 Ab treatment. Scale bar: 100 μm. Data represent mean ± SD. * *p* < 0.05; ** p < 0.01; *** p < 0.001; n.s. non-significance, Student's *t*-test.

We first performed a syngeneic mouse model by subcutaneously injecting LLC cells into immunocompetent C57BL/6 mice and measuring the tumor growth and immune cell profiling with or without intravenous (i.v.) injection of CHI3L1 nAbs (5 μg/g) once every three days, starting on day 3 until day 12 (**Figure 5A**). As shown in **Figure 5B-D**, tumor growth measured by tumor volume, tumor size, and tumor weight was significantly reduced after nCHI3L1 Ab treatment as compared to the IgG control. Importantly, the concentration of CHI3L1 in the plasma from nCHI3L1 Ab-treated mice was maintained at a normal range until nCHI3L1 Ab treatment was stopped, while elevated CHI3L1 plasma level was observed in the control group (**Figure 5E**). The profile of tumor infiltrating immune cells was analyzed by FACS and the results revealed that the percentage of immunosuppressive CTLA4^+^ or PD-1^+^ on CD8^+^ T cells and CD4^+^CD25^+^Foxp3^+^ Tregs was significantly reduced in nCHI3L1 Ab treatment group compared to that in the IgG group (**Figure 5F**). Notably, therapeutic efficacy of nCHI3L1 Abs was better than that of anti-CTLA-4 antibody (α-CTLA-4) in LLC subcutaneous tumor growth model (Figure S6).

Next, we utilized syngeneic allograft tumor models by orthotopically injecting LLC cells into the left lobe of lung in C57BL/6 mice. The mice were treated with IgG control or nCHI3L1 Abs as illustrated in **Figure 5G**. On day 20, the mice were sacrificed, and the lungs were harvested. Lung orthotopic model, by which LLC cells inoculated percutaneously into the left lateral thorax, was measured as the growth of primary tumor while the tumor nodules at the right lungs of injected mice were considered as metastatic lesions. Histologic examination of tumors in H&E-stained sections clearly showed a large area of LLC primary tumor nodules in mouse lungs treated with IgG antibody, while only a small area of primary tumors was detected in nCHI3L1 Ab-treated mouse lungs (**Figure 5H**). Notably, the nCHI3L1 Ab treatment attenuated tumor metastases to the contralateral lungs (**Figure 5I**). The body weight, serum biochemical markers and major organ of treated mice examined revealed no significant adverse effects upon nCHI3L1 Ab treatment (**Figure 5J-L**). These findings demonstrated that nCHI3L1 Ab treatment effectively inhibited lung tumor growth and metastasis *in vivo* and improved T cell activation without apparent adverse effects.

### nCHI3L1 Abs inhibit tumor growth and metastasis, and elicit an anti-tumor immunity in orthotopic pancreatic tumor models

**Figure 6:**
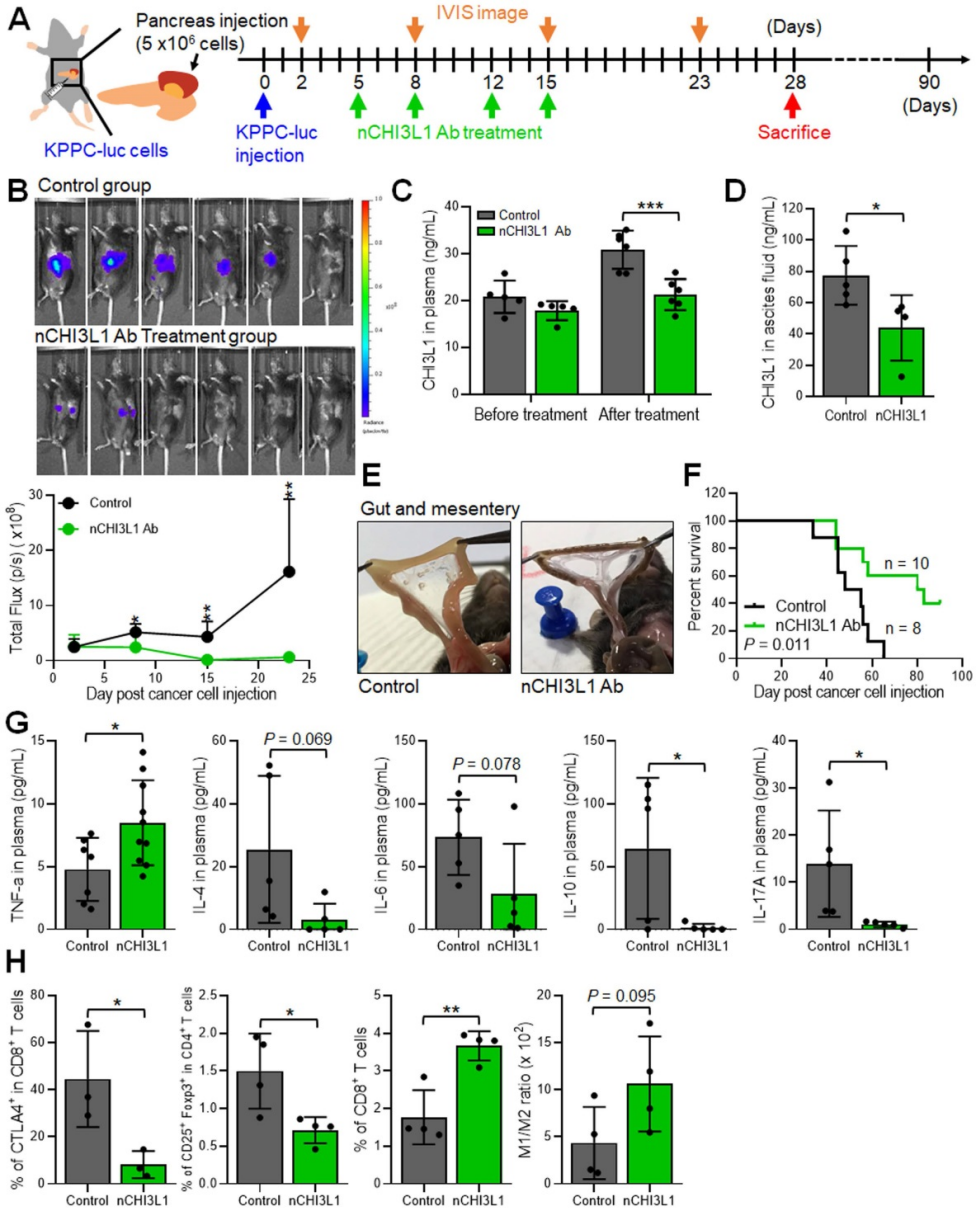
** nCHI3L1 Ab inhibits tumor growth and metastasis and elicits an anti-tumor immunity in orthotopic pancreatic tumor model.** (**A**) The scheme showing the pancreas orthotopic model using KPPC-luc cells in *C57BL/6* mice. (**B**) The nCHI3L1 Ab treatment decreased the tumor signal according to IVIS imaging (*Upper*) and quantitative analysis (*Lower*). (**C**, **D**) Elevated concentration of CHI3L1 in plasma (C) or ascites (D) was observed in the control group, while maintained at the normal range CHI3L1 level from nCHI3L1 Ab-treated mice. (**E**) nCHI3L1 Ab treatment attenuated tumor metastases to the gut and mesentery. (**F**) Survival curves were measured in pancreatic cancer orthotopic mouse model. (**G**) nCHI3L1 Ab treatment reprogrammed to Th1 inflammation measured by cytometric bead array of Th1/Th2/Th17 markers in PBMC. (**H**) Tumor-infiltrating CTLA-4^+^ on CD8^+^ cells, Treg cells total CD8 T cells and macrophages M1/M2 ratio were measured by FACS. Data represent mean ± SD. * *p* < 0.05; ** p < 0.01; *** p < 0.001, Student's *t*-test.

To further explore whether nCHI3L1 Abs exert treatment potential in additional aggressive cancer, we performed PDAC orthotopic model using luciferase stable line derived from pancreas-specific *Kras^G12D/+^*; *Trp53^fl/fl^; Pdx-Cre* mice (KPPC-luc cells). The treatment protocol is described in **Figure 6A**. The nCHI3L1 Ab treatment decreased the tumor signal according to IVIS analysis (**Figure 6B**). Additionally, nCHI3L1 Ab treatment reduced CHI3L1 level in plasma and ascites compared to IgG group (**Figure 6C**-**D**). Of note, nCHI3L1 Ab treatment attenuated tumor metastases to the gut and the mesentery (**Figure 6E**). Importantly, nCHI3L1 Ab treatment prolonged mouse survival (**Figure 6F**). FACS results revealed that nCHI3L1 Ab treatment reprogrammed to Th1 response in the peripheral blood mononuclear cells (PBMCs) collected from endpoint (**Figure 6G**). Similar Th1 response results were observed in the ascites (data not shown). Tumor infiltrating immune cell profile analyses also revealed the reduction of immuno-suppressive CTLA-4^+^CD8^+^ cells and CD4^+^CD25^+^Foxp3^+^ Tregs, whereas the percentage of tumor infiltrating cytotoxic CD8^+^ T cells and the ratio of M1/M2 macrophages increased in nCHI3L1 Ab treatment group (**Figure 6H**). Collectively, these results suggested that nCHI3L1 Abs could be a novel approach for controlling PDAC tumor growth and progression.

### nCHI3L1 Abs show stronger inhibition of tumor growth and metastasis than bevacizumab in CRC tumor models

**Figure 7:**
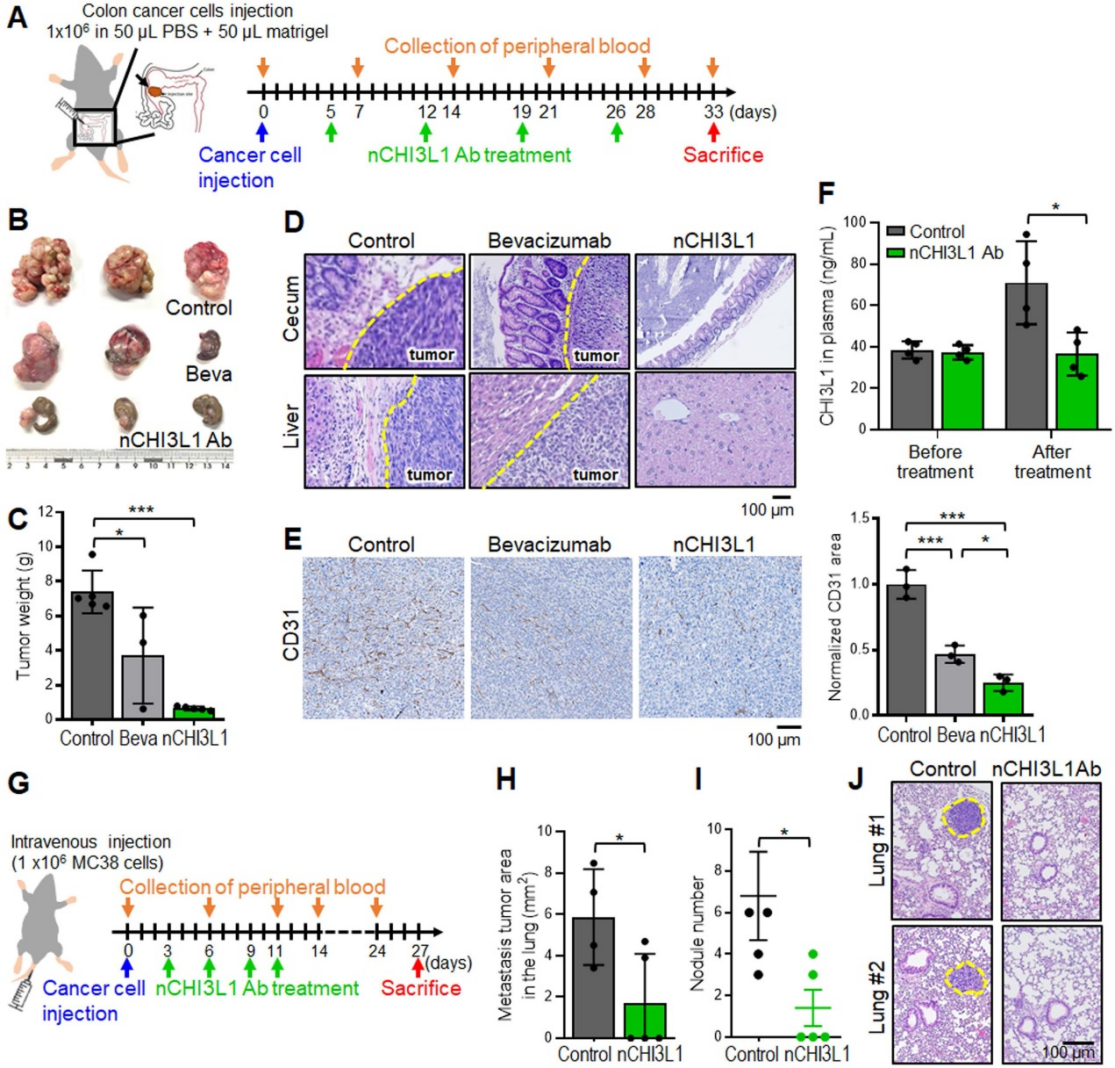
** nCHI3L1 Ab shows stronger inhibition of tumor growth and metastasis than bevacizumab in CRC tumor models.** (**A**) Colon cancer orthotopic model with MC-38 injected into the cecum in *C57BL/6* mice was treated as shown in the scheme. (**B, C**) The colon images (B) and tumor weight (C) clearly showed large primary tumor nodules in the IgG control group and some tumor nodules in the bevacizumab mice group, while tumor weights were significantly reduced in nCHI3L1 Ab treated mice group. (**D**) H&E-stained sections of primary cecum tumors (*Upper*) and metastatic tumors in liver (*Lower*). Scale bar: 100 μm. (**E**) IHC images (*Left*) and quantification (*Right*) of CD31-positive areas in primary tumor specimens from IgG control, nCHI3L1 Ab-treated, and bevacizumab-treated mice group. Scale bar: 100 μm. (**F**) The concentration of CHI3L1 in the plasma from IgG control and nCHI3L1 Ab treated mice measured on the day mice were sacrificed. (**G**) Schematic presentation of tail-vein injection experimental metastasis model of MC-38 in *C57BL/6* mice. (**H, I**) Metastasis tumor area (H) and tumor nodule number (I) in IgG control mice or nCHI3L1 Ab treated mice. **(J)** Representative H&E-stained sections lung metastatic tumors in mouse of IgG control (*Left*) or nCHI3L1 Ab (*Right*) treatment group. Scale bar: 100 μm. Data represent mean ± SD. * *p* < 0.05; ** p < 0.01; *** p < 0.001, one-way ANOVA test (C, E); Student's *t*-test (F, H, I).

CRC is also an aggressive cancer for which only bevacizumab is FDA approved as a first- and second-line VEGF-targeted therapeutic antibody 33. Importantly, CHI3L1 and VEGF synergistically promote angiogenesis in cancers 11, 34, 35. We next sought to compare the efficacy of nCHI3L1 Abs and bevacizumab in CRC orthotopic model in which MC38 cells were inoculated onto the cecum, and the presence of primary tumors and distant metastasis can be macroscopically assessed and microscopically examined for liver metastasis (**Figure 7A**). Strikingly, nCHI3L1 Ab treatment not only significantly reduced the primary tumor growth (**Figure 7B**-**C**) but also decreased tumor metastases to the liver compared with the control group or even with the bevacizumab group (**Figure 7D**). Moreover, we examined whether CD31 endothelial cells marker was decreased in tumor allograft derived from CRC *in vivo* models. IHC analysis revealed that CD31 signal was decreased in nCHI3L1 Ab treatment group compared to control group (**Figure 7E**). Notably, mice group injected with nCHI3L1 Ab showed less CD31 signal than those from bevacizumab group. Additionally, the ELISA data showed a positive correlation of plasma CHI3L1 level with tumor volume in treated mice (**Figure 7F**). These results showed that nCHI3L1 Ab treatment exerted anti-tumor and anti-angiogenic effects *in vivo*.

Furthermore, we employed the tail-vein injection model to confirm the anti-metastasis effect of nCHI3L1 Abs in CRC mouse model (**Figure 7G**). Large tumor nodules in the lung of the control mice were observed, while nCHI3L1 Ab treatment reduced the size and number of metastatic tumor nodules (**Figure 7H-J**), suggesting that nCHI3L1 Abs suppressed colon tumor metastases to the lung. Collectively, the results from three preclinical models of NSCLC, PDAC and CRC demonstrated the anti-tumor effects and immunomodulatory functions of nCHI3L1 Abs.

### nCHI3L1 Abs decrease intratumoral CHI3L1 expression and induce infiltration of CD8^+^ T cells and M1 macrophages into the TME of LLC tumor

**Figure 8:**
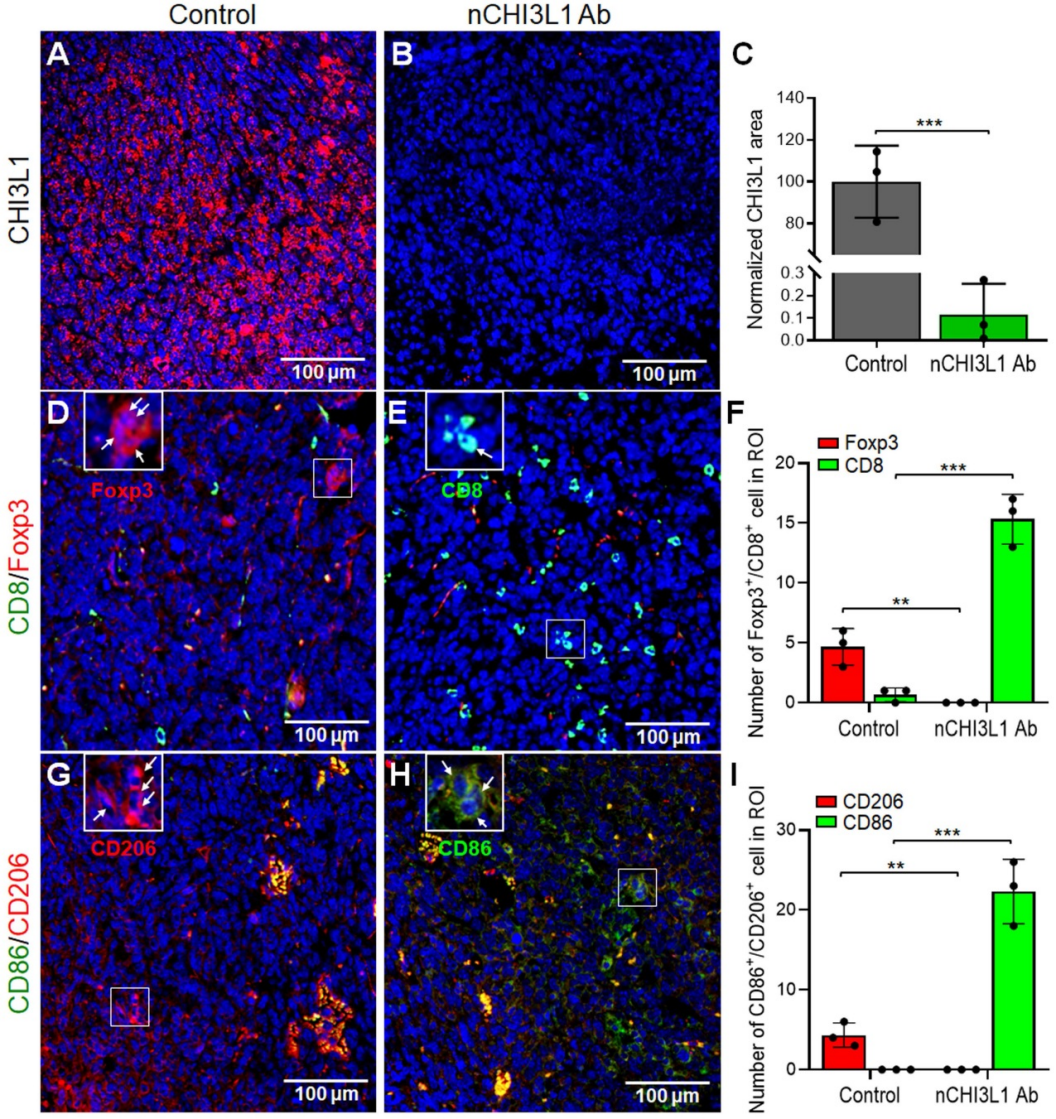
** nCHI3L1 Ab downregulates intratumoral CHI3L1 expression and modulates tumor infiltrating immune cells *in vivo*.** (**A-C**) IF-IHC was performed on tissue sections of LLC lung tumor tissues which showed abundant intratumoral CHI3L1 signals in LLC tumors of IgG control mice (A). nCHI3L1 Ab treatment largely reduced the intratumoral CHI3L1 expression (B, C). (**D-F**) The representative IF-IHC images (D, E) and quantification (F) of distribution of CD8^+^ T cells (green) and Treg cells (red) in IgG control group (D) and nCHI3L1 Ab treatment group (E). (**G-I**) Distribution (G, H) and quantification (I) of M1 (green) and M2 (red) macrophages in IgG control group (G) and nCHI3L1 Ab treatment group (H). Enlarged images shown in insets of the representative regions are numbered as indicated. Blue fluorescence represents the nucleus staining. Scale bars: 100 μm. Each quantitative circle is an average of immunoreactive positive cells per three regions of interest (ROI, 200 x 200 μm). Data are representative of three tumor sections. * *p* < 0.05; ** p < 0.01; *** p < 0.001, Student's *t*-test.

To further proof that targeting CHI3L1 by nCHI3L1 Abs resulted in decrease of CHI3L1 expression to elicit an immune-stimulatory TME, multi-color IF-IHC was performed on the LLC tumor sections. Strikingly, nCHI3L1 Ab treatment largely reduced the intratumoral CHI3L1 expression compared to the IgG group (**Figure 8A**-**C**). Of note, nCHI3L1 Ab treatment promoted higher CD8^+^ T cells to FOXP3^+^ Tregs (CD8^+^/Treg) ratio infiltrating into the inner tumor regions as compared to the IgG group (**Figure 8D**-**F**). Importantly, less M1 macrophages (CD86) to M2 (CD206) (M1/M2) ratio was found in the inner region of tumor sections in the IgG group (**Figure 8G, 8I**; Figure S7A). Conversely, there was higher M1/M2 ratio in the tumor center after nCHI3L1 Ab treatment (**Figure 8H, 8I**; Figure S7B). These IF-IHC results demonstrated novel findings that nCHI3L1 Ab therapies reduce CHI3L1 accumulation and shaped an anti-tumor immunity TME to suppress tumor growth and metastasis.

### Rab37^+^CHI3L1^+^ cells are associated with M2 tumor associated macrophages in lung cancer patients

**Figure 9:**
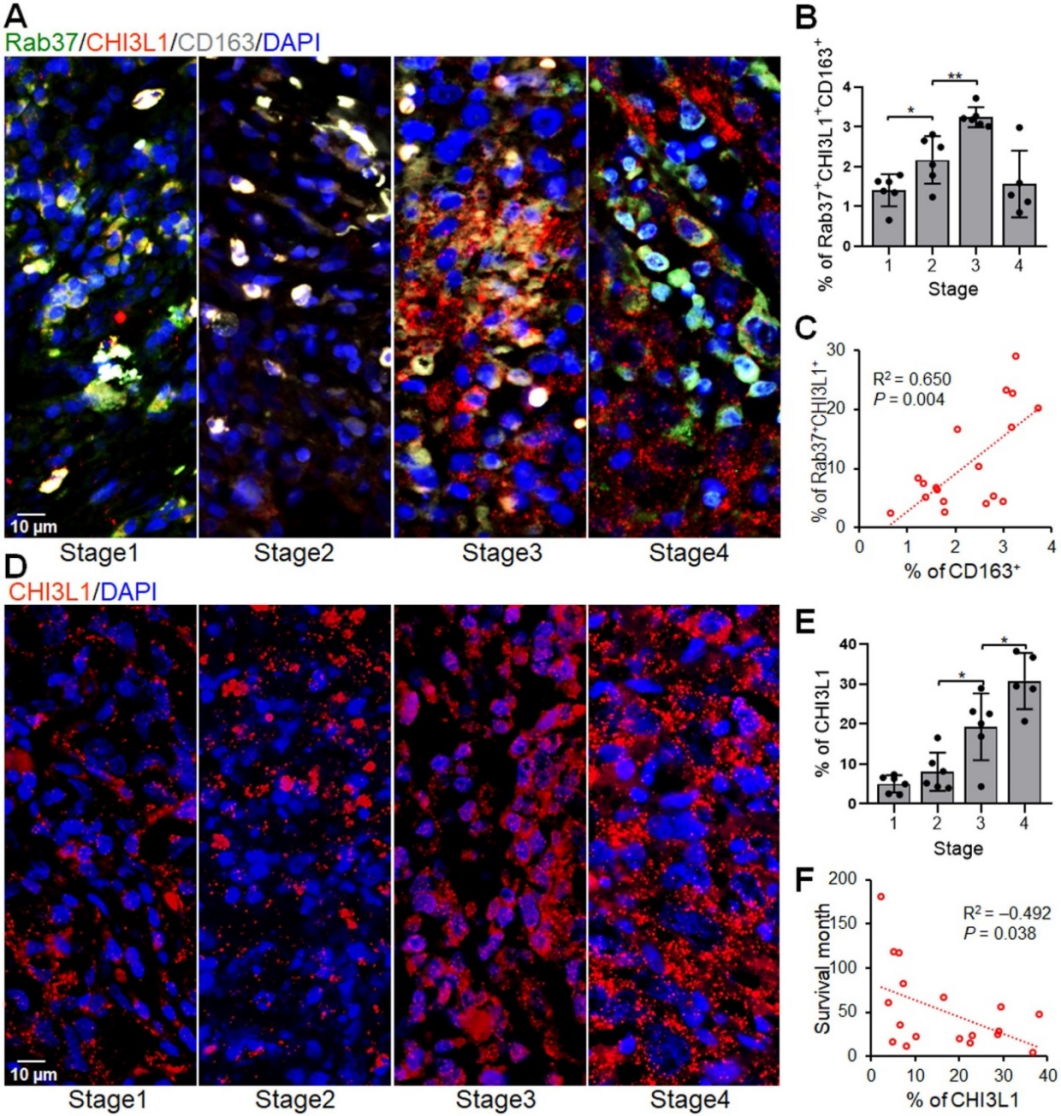
** Intratumoral Rab37^+^CHI3L1^+^ areas are enriched with CD163^+^ M2 TAMs in advanced stage lung cancer patients.** (**A, B**) The representative multiplex IF-IHC images (A) and proportion of infiltrated Rab37^+^CHI3L1^+^CD163^+^ M2 TAMs (B) correlated with the tumor progression stages (*n* = 23) (stage I *vs*. stage II, *p* < 0.05; stage II *vs*. stage III, *p* < 0.01, one-way ANOVA test.). (**C**) Scatter plot showing the correlation between Rab37^+^CHI3L1^+^ cells with CD163^+^ cells in lung cancer patients (*n* = 18). Pearson correlation coefficient, R square and *P*-value are shown. (**D, E**) The representative IF-IHC images (D) and proportion of intratumoral CHI3L1 stained signals (E) correlated with the tumor progression stages (*n* = 23) (stage II *vs*. stage III, *p* < 0.05; stage III *vs*. stage IV, *p* < 0.05, one-way ANOVA test.). (**F**) Scatter plot showing the inverse correlation between intratumoral CHI3L1 signals with survival times in lung cancer patients (*n* = 18). Pearson correlation coefficient, R square and *p*-value are shown. Each quantitative circle represents data from one patient and is an average of immunoreactive positive cells per three ROIs (450 x 450 μm).

The IF-IHC results in mouse allograft tumors prompted us to verify if tumors from cancer patients with high expression of both Rab37 and CHI3L1 (Rab37^+^CHI3L1^+^) also foster an immunosuppressive TME. We examined whether the infiltrating M2 tumor associated macrophages (M2 TAM, CD163^+^) were enriched in the area of Rab37^+^CHI3L1^+^ cells in tumor specimens from lung cancer patients. Multiplex IF-IHC of Rab37, CHI3L1 and CD163 was performed on 23 tumor specimens. Representative images of patients with stage 1, 2, 3, or 4 cancer are shown in **Figure 9A**. The tumor infiltrating Rab37^+^CHI3L1^+^ cells were highly enriched with CD163^+^ M2 TAMs in the tumor sections from patients with advanced tumor stages, whereas early staged tumors displayed a low abundance of infiltrating Rab37^+^CHI3L1^+^CD163^+^ M2 TAMs and scattered intratumoral CHI3L1 (**Figure 9A-B**). Of note, tumors from stage 4 patients mostly expressed CHI3L1 in the extracellular compartment, therefore there were less intracellular colocalized Rab37^+^CHI3L1^+^CD163^+^ cells (**Figure 9A-B**). Moreover, quantitative analysis indicated that the regions enriched in Rab37^+^CHI3L1^+^ cells positively correlated with high infiltration of CD163^+^ M2 TAMs (R square = 0.650, *P* = 0.004, *n =* 18) (**Figure 9C**). Notably, patients with high intratumoral CHI3L1 staining signals negatively correlated with survival time (R square = -0.432, *P* = 0.038, *n =* 18) (**Figure 9D-F**). Together, these clinical results demonstrated for the first time that tumors from advanced lung cancer patients with poor survival was characterized by a distinct immunosuppressive TME with a high level of stromal CHI3L1^+^Rab37^+^ associated with tumor infiltrating M2 macrophages.

### High plasma level of CHI3L1 correlates with poor prognosis of NSCLC, PDAC and CRC patients

**Figure 10:**
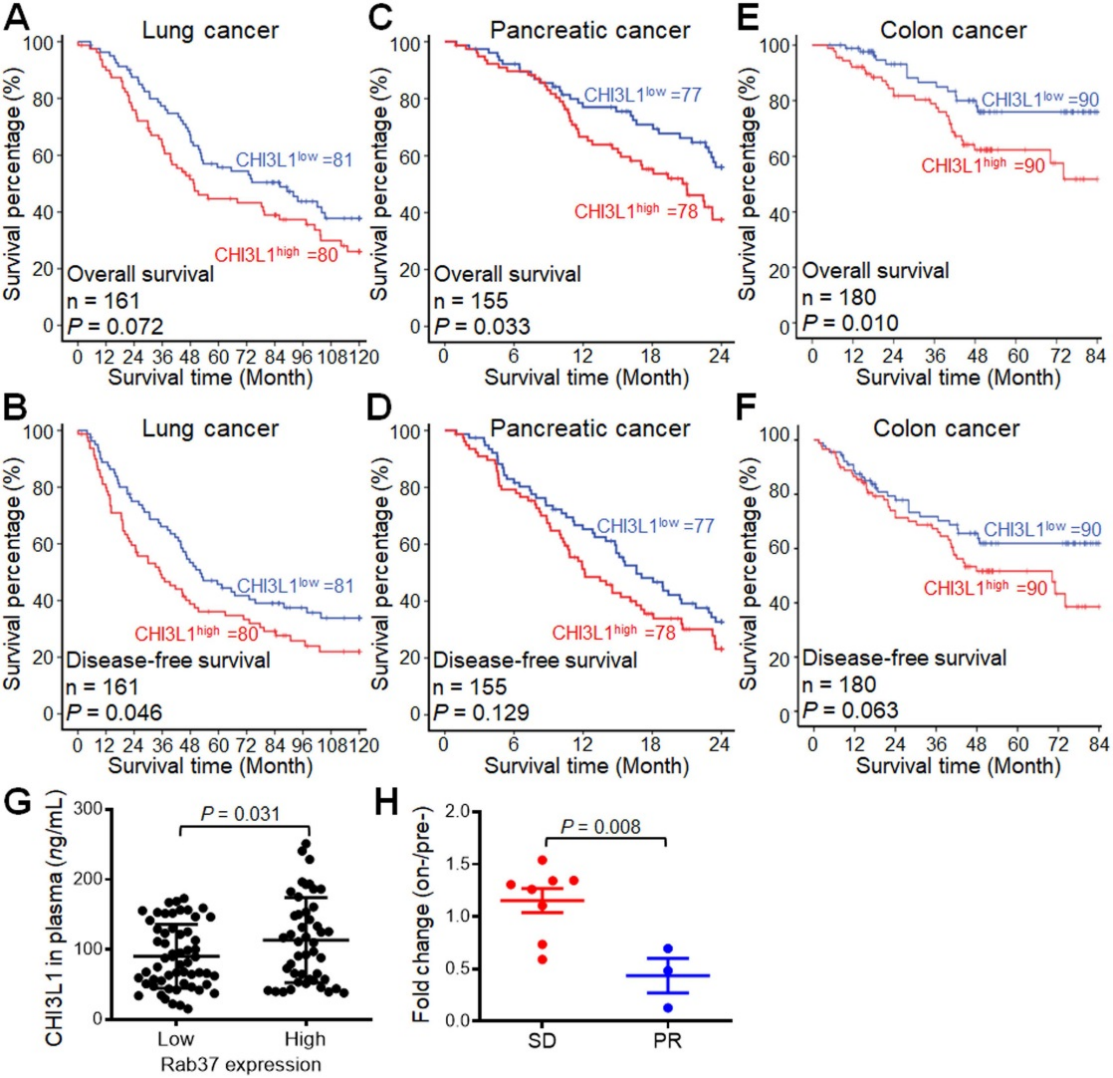
** High plasma level of CHI3L1 correlates with poor prognosis and therapeutic efficacy in cancer patients.** (**A, B**) Kaplan-Meier method of overall survival (A) or disease-free survival (B) of lung cancer patients (*n* = 161) with high plasma level of CHI3L1 showing significantly poorer survival than patients with low level ones. (**C, D**) Pancreatic cancer patients (*n* = 155) with high plasma level of CHI3L1 showing significantly poorer overall survival (C) or disease-free survival (D). (**E, F**) Colon cancer patients (*n* = 180) with high plasma level of CHI3L1 showing significantly poorer overall survival (E) or disease-free survival (F). *P* values determined using log-rank test. (**G**) Dot plot showing the correlation between plasma level of CHI3L1 with tumoral expression of Rab37 in lung cancer patients. The Rab37 tumor IHC is divided into low and high groups as indicated in the X-axis, while the plasma level of CHI3L1 is indicated in the Y-axis. One dot represented one patient (*n* = 103). *P* value was calculated by 2-tailed t test. (**H**) Changes of CHI3L1 level in plasma from pretreatment to on-treatment of α-PD-1 blockade of lung cancer patients were measured. Fold changes of CHI3L1 levels (on-/pre) were analyzed. Lung cancer patients were grouped based on their clinical responses (SD: stable disease, red, *n* = 8; PR: partial response, blue, *n* = 3).* P* value was calculated by 2-tailed t test.

Our ELISA data in animal models indicated a positive correlation of plasma CHI3L1 level and tumor size (**Figure 5E, 6C**, **7F**). In addition, our clinical IF-IHC results showed that intratumoral CHI3L1^+^ expression was associated with cancer progression in terms of tumor staging (**Figure 9E**). Therefore, we interrogated whether plasma level of CHI3L1 correlated with prognosis of cancer patients. ELISA results showed that increased plasma CHI3L1 concentration correlated with poorer overall survival and disease-free survival of our in-house cohort of NSCLC (*n =* 161), PDAC (*n =* 155) and CRC (*n =* 180) patients **(Figure 10A-F** and Table S3**)**. We defined patient with high CHI3L1 expression (CHI3L1^high^) as the expression percentage greater than the mean (61.15 *n*g/mL for NSCLC; 89.75 *n*g/mL for PDAC; 105.80 *n*g/mL for CRC). Next, multivariate Cox regression analysis revealed that NSCLC, PDAC or CRC patients with CHI3L1^high^ expression profile showed significantly high risk of death even after adjusting for other clinical parameters (**Table 1**). The high level of plasma CHI3L1 accompanied by poor prognosis of cancer patients suggesting that CHI3L1 is a biomarker of cancer progression as well as a therapeutic target in NSCLC, PDAC and CRC.

Moreover, the relationship between tumoral Rab37 expression and circulation CHI3L1 level has never been examined in human cancer patients. Therefore, we performed tumor IHC with Rab37 and ELISA of plasma CHI3L1 from 103 PDAC patients whose tumor specimen and plasma samples were both available. Association study showed that plasma CHI3L1 levels correlated with tumor tissue Rab37 expression (*P* = 0.008, **Figure 10G**), validating for the first time that Rab37 mediates CHI3L1 secretion in PDAC clinical model.

### Change in the plasma CHI3L1 level reflects the treatment response to immunotherapy in NSCLC patients

These clinical findings highlighted that CHI3L1 can be a promising marker for monitoring outcome status. Next, we further studied whether plasma level of CHI3L1 was associated with therapeutic efficacy of α-PD-1 treatment in lung cancer patients. We performed ELISA assay on plasma samples derived from 11 lung cancer patients receiving immunotherapy whose sequential plasma samples were available. As shown in **Figure 10H**, patients with poor clinical responses (SD, stable disease) correlated with higher increase in plasma CHI3L1 levels during immunotherapy (on-/pretreatment; median ratio, 1.21-fold) as compared to those with partial response (PR; 0.42-fold, *P* < 0.01). These ELISA data implicated that an increase in plasma CHI3L1 level during immunotherapy was associated with poor response to treatment in lung cancer patients.

## Discussion

**Figure 11:**
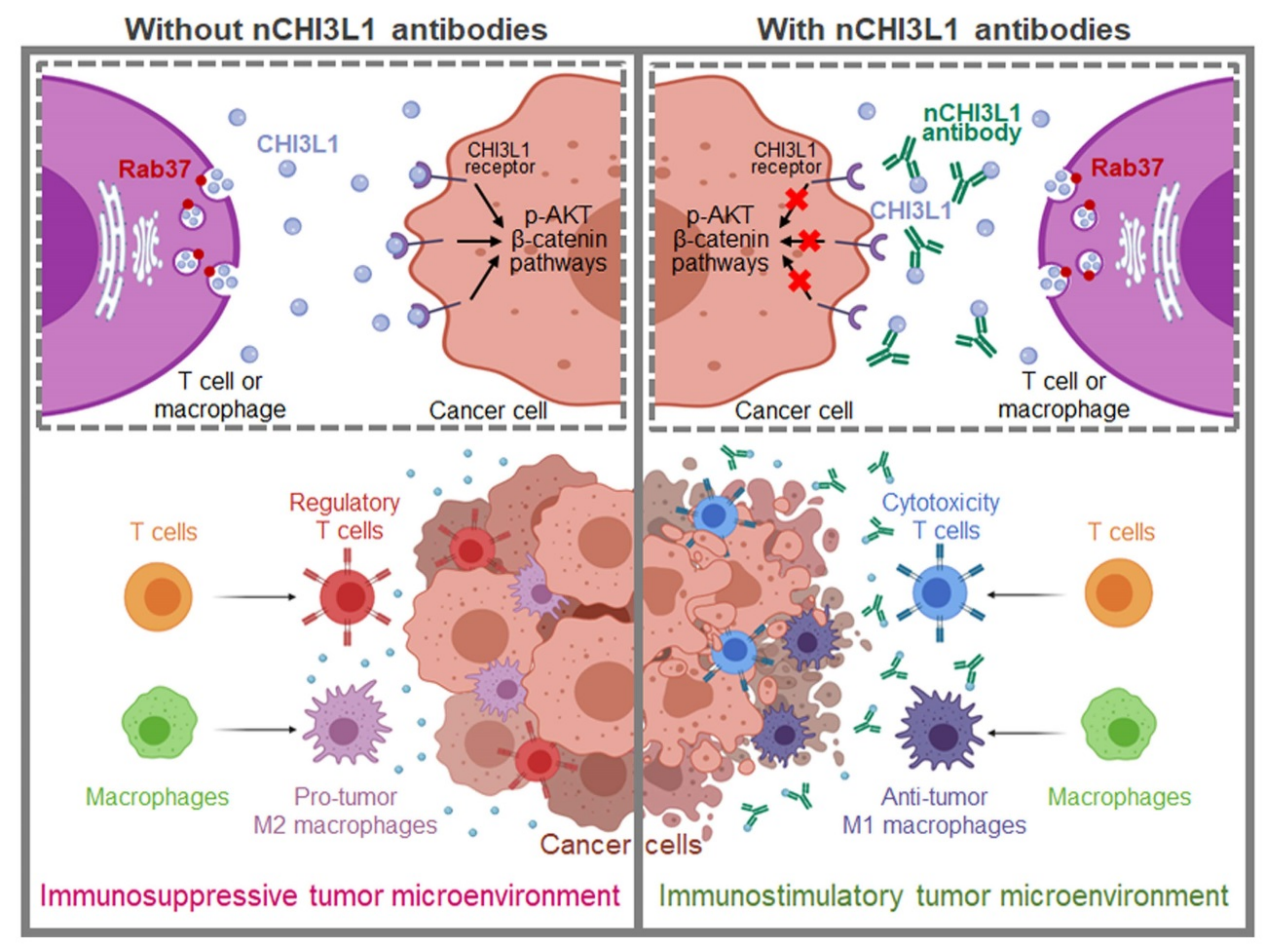
** Schematic model of Rab37-mediated exocytosis of CHI3L1 protumor protein in T cells or macrophages to foster an immunosuppressive tumor microenvironment that is blocked by CHI3L1 neutralizing antibodies through activating anti-tumor immune response and reducing cancer oncogenic signals.** This study provides cell, animal and clinical evidence that exocytosis of chitinase 3-like-1 (CHI3L1) protumor protein mediated by Rab37 vesicles in T cells and macrophages drives tumor progression and immunosuppressive microenvironment (*Left*). These immunosuppressive tumor microenvironment and aggressive tumor phenotype are blocked by CHI3L1 neutralizing antibodies through shifting M2 to anti-tumor M1 macrophages, decreasing regulatory T cells/cytotoxic T cells ratio and attenuating oncogenic cancer signaling to reduce tumor growth and metastases (*Right*).

As a protumor secretion factor, CHI3L1 has been shown to regulate key processes within TME including inflammation, cell proliferation, differentiation, angiogenesis and remodeling of the extracellular matrix thus promotes tumor progression 4, 5. Here, we reveal novel findings that Rab37 mediates CHI3L1 secretion in T cells and macrophages, the major constituent cells in TME, to promote M2 macrophage polarization and higher FOXP3^+^ Tregs to CD8^+^ T cells ratio in the tumor region, ultimately establishing an immunosuppressive TME. In addition, secreted CHI3L1 binds to the receptor on cancer cells and macrophages to activate AKT, β-catenin and NF-κB signaling pathways to promote tumor growth and metastasis. These immunosuppressive TME and aggressive tumor phenotype are blocked by CHI3L1 nAbs through shifting M2 to anti-tumor M1, decreasing Tregs/CD8^+^ T ratio and attenuating oncogenic cancer signaling to reduce tumor growth and metastases. The decreased plasma level of CHI3L1 in nCHI3L1 Abs-treated mouse groups and those patients responding to anti-PD-1/PD-L1 immunotherapy strongly suggest that CHI3L1 can be a therapeutic target and a biomarker for disease monitoring (**Figure 11**).

CHI3L1 has been previously shown to localize to the Golgi apparatus and dynamin II-mediated intracellular vesicles in macrophages 36, 37. However, the secretion mechanism of CHI3L1 has never been elucidated. We demonstrate for the first time that Rab37 mediates intracellular vesicle trafficking and exocytosis of CHI3L1 in a GTPase-dependent manner not only in macrophages but also in T cells. We and others have previously shown that Rab37 regulates vesicle trafficking from the *trans*-Golgi network to the plasma membrane 24, 38. Recently, Rab37 is found to be a key regulator of autophagosome formation 39. Of note, Kzhyshkowska and colleagues reported that chitinase 3-like-2 (CHI3L2), a family member of CHI3L1 but not a glycoprotein, is localized to the *trans*-Golgi network around the nuclear area in macrophages 34. CHI3L2 is sorted by stabilin-1 to the secretory lysosome then secreted 27. Importantly, CHI3L1 is found to be packaged into the extracellular vesicles derived from macrophages 16. Rab37 belongs to the same exocytic pathway as Rab27a and Rab27, which involve the biogenesis of extracellular vesicles 40, 41. Thus, it is interesting to interrogate whether Rab37-mediated CHI3L1 secretion is also dependent on alternative pathways such as extracellular vesicles, secretory lysosome or secretory autophagy. Nevertheless, our findings shed new light on the secretion mechanism of CHI3L1 by Rab37 small GTPase in macrophages and T cells.

Our IF-IHC results of LLC allograft in mouse and tumor specimens from lung cancer patients demonstrated a strong expression of CHI3L1 in macrophages, CD4^+^ T cells and CD8^+^ T cells, suggesting that macrophages and T cells are an important source of CHI3L1 in tumors. In addition, our nCHI3L1 Ab treatment reduced M2 macrophage polarization and Treg differentiation *in vitro* and promoted infiltration of M1 macrophages and CD8^+^ T cells *in vivo.* Alteration in M2-like differentiation of macrophages and imbalance of Th1/Th2 effector responses in CHI3L1 knockout mice 2, 13 further support our notion that CHI3L1 was mainly derived from M2 macrophages and Th2 cells. Additionally, CHI3L1 is highly expressed and produced by specialized M2a macrophages to drive Th2-mediated differentiation in a chronic inflammatory skin disorder 13. These studies suggest that the T cells and macrophages secrete high level of CHI3L1 during inflammation and in TME. However, we cannot exclude the possibility that intratumoral CHI3L1 expression may originate from multiple cell types, for example, neutrophils, cancer cells, and cancer-associated fibroblasts 12, 42.

It has been suggested that cancer cells express low levels of CHI3L1 and its expression depends on the stage of the cancer 4, 43. In this study we show that exogenous addition of recombinant CHI3L1 protein promoted the AKT and β-catenin pathways in cancer cells, suggesting that stromal-derived CHI3L1 may affect cancer cells in a paracrine manner within the TME. Recently, we showed that Rab37 mediates IL-6 secretion to upregulate STAT3 signaling 23. Importantly, IL-6 regulates CHI3L1 secretion in macrophage 44 and STAT3 binds to the promoter of CHI3L1 to induce its transcription 45. It is intriguing to speculate that Rab37/IL-6/CHI3L1 may form a feedback loop to elicit an immunosuppressive TME. Notably, CHI3L1 is also upregulated by growth factors such as androgens/androgen receptor in prostate cancer and constitutive estrogen receptor alpha in breast cancer 46, 47. Further research is required to obtain a comprehensive picture of the cancer-intrinsic CHI3L1 regulated by cytokines or growth factors involved in the cross talk between the stromal cells and cancer cells in TME.

Our clinical results showed that NSCLC patients with an increase in plasma CHI3L1 level were significantly associated with poor prognosis or therapeutic resistance to anti-PD-1/PD-L1 immunotherapy. Moreover, our nCHI3L1 Abs exhibited comparable or even better *in vivo* anti-tumor efficacy as compared with α-CTLA4 or bevacizumab (anti-VEGF antibody) treatment. CHI3L1 have been shown to work synergistically with VEGF to promote endothelial cell angiogenesis and tumor progression through FAK and ERK1/2 activity 10, 48. We hypothesized that nCHI3L1 Abs can complement the immunotherapy against NSCLC or even PDAC and CRC tumors.

Notably, the in-house developed nCHI3L1 Abs exerted dual functions in reducing tumor growth/metastases and activating an immunostimulatory in syngeneic orthotopic lung, pancreas and colon tumor models. These results highlight nCH3L1 Abs that can simultaneously target both cancer cells and tumor microenvironment. We and others have shown that CHI3L1 is a pleotropic protumor secretion factor that promotes cancer cell growth and metastasis *via* AKT, MAPK, β-catenin, NF-κB and MMP signaling 17, 19, 20, 32, 49. CHI3L1 also induces a number of pro-tumorigenic cytokines such as CCL2, CXCL2 and IL-8 9, 50, 51, and mediates Th2 immune response to activate M2 macrophages and increase Treg/CD8 ratio 2, 13. Considering the multiple roles of CHI3L1 in tumorigenesis and low basal level in normal epithelial cells, the direct neutralization of secreted intratumoral CHI3L1 will inhibit tumor progression.

In summary, using imaging and biochemical analyses, we systematically investigated the trafficking mode of protumor secretion glycoprotein CHI3L1 in *ex vivo* isolated macrophages and splenic T cells as well as respective cell line models, and identified Rab37 as a critical mediator for intracellular trafficking and exocytosis of CHI3L1. Functional studies revealed the pathological significance of CHI3L1 in driving both tumor progression and immunosuppressive TME. Our newly developed nCHI3L1 Abs exerted dual anti-tumor functions by inhibiting AKT, β-catenin and NF-κB oncogenic signaling in cancer cells and modulating an immunostimulatory TME with more tumor infiltrating M1/M2 and CD8^+^ T/Treg ratios in lung, pancreas and colon orthotopic syngeneic mouse models. This translational potential is reinforced and broadened by the marked correlation of tissue and circulating CHI3L1 with prognosis or treatment response of immunotherapy in NSCLC, PDAC and CRC. Therefore, CHI3L1 represents an attractive therapeutic target and biomarker predicting resistance to anti-PD-1/PD-L1 therapy and warrants further investigation to benefit patients with different types of cancer.

## Supplementary Material

Supplementary figures and tables.

## Figures and Tables

**Table 1:** Cox regression analysis of risk factors for cancer-related death in lung, pancreatic and colon cancer patients.

Characteristics		A. Lung cancer patients				B. Pancreatic cancer patients				C. Colon cancer patients			
		Univariate analysis		Multivariate analysis		Univariate analysis		Multivariate analysis		Univariate analysis		Multivariate analysis	
		HR ^A^ (95% CI ^B^)	*P*-value ^C^	HR (95% CI)	*P*-value	HR (95% CI)	*P*-value	HR (95% CI)	*P*-value	HR (95% CI)	*P*-value	HR (95% CI)	*P*-value
**CHI3L1**	CHI3L1^ low^	1		1		1		1		1		1	
**expression** ^D^	CHI3L1^ high^	1.41 (0.961-2.066)	**0.078**	1.56 (1.056-2.293)	**0.025**	1.66 (1.038-2.665)	**0.034**	1.62 (1.013-2.606)	**0.044**	2.21 (1.188-4.102)	**0.012**	2.20 (1.177-4.100)	**0.013**
**Age**	< 65 year-old	1		- ^F^		1		-^ F^		1		-^ F^	
	≥ 65 year-old	0.92 (0.628-1.357)	0.685	- ^F^	- ^F^	1.42 (0.888-2.256)	0.144	-^ F^	- ^F^	1.80 (0.957-3.373)	0.068	-^ F^	- ^F^
**Gender**	Female	1		- ^F^		1		-^ F^		1		-^ F^	
	Male	1.42 (0.968-2.080)	0.073	- ^F^	- ^F^	1.04 (0.654-1.651)	0.872	-^ F^	- ^F^	0.84 (0.459-1.536)	0.571	-^ F^	- ^F^
**Stage**	Stage I-II	1		1		1		1		1		1	
	Stage III-IV	1.98 (1.350-2.912)	**< 0.001**	1.13 (0.624-2.042)	0.689	1.57 (0.922-2.685)	0.096	1.51 (0.879-2.583)	0.136	3.61 (1.831-7.123)	**< 0.001**	0.98 (0.183-5.203)	0.977
**T status** ^E^	Stage 1-2	1		1		-		-^ F^		1		1	
	Stage 3-4	1.75 (1.072-2.853)	**0.025**	1.33 (0.771-2.285)	0.307	-	-	-^ F^	- ^F^	3.66 (1.445-9.269)	**0.006**	2.13 (0.794-5.692)	0.134
**N status** ^E^	N0	1		1		-		-^ F^		1		1	
	≥ N1	2.11 (1.432-3.110)	**< 0.001**	1.79 (1.032-3.088)	**0.038**	-	-	-^ F^	- ^F^	3.26 (1.711-6.193)	**< 0.001**	1.43 (0.334-6.136)	0.629
**M status** ^E^	M0	1		1		1		1		1		1	
	≥ M1	4.46 (2.227-8.939)	**< 0.001**	3.14 (1.508-6.527)	**0.002**	1.50 (0.903-2.499)	0.117	1.37 (0.818-2.294)	0.231	3.02 (1.681-5.430)	**< 0.001**	1.96 (0.975-3.948)	0.059
**Recurrence**	No	1		- ^F^		1		- ^F^		1		1	
	Yes	1.06 (0.685-1.629)	0.803	- ^F^	- ^F^	0.90 (0.567-1.434)	0.661	- ^F^	- ^F^	3.67 (2.027-6.645)	**< 0.001**	2.47 (1.314-4.641)	**0.005**

A: HR: Hazard ratio. B: CI: Confidence interval. C: Bold values indicate statistical significance (p < 0.05). D: CHI3L1^ high^, high expression; CHI3L1^ low,^ low expression for CHI3L1. Lung patient with CHI3L1 protein concentration ≥ 61.15 *n*g/mL was defined as high expression. Pancreatic patient with CHI3L1 protein concentration ≥ 89.75 *n*g/mL was defined as high expression. Colon patient with CHI3L1 protein concentration ≥ 105.80 *n*g/mL was defined as high expression. E: T status: primary tumor; N status: lymph node metastasis; M status: distant metastasis. F: The variables without significant HR in the univariate analysis were not included in the multivariate analysis.
