# Supplementary material for: Targeting protumor factor chitinase-3-like-1 secreted by Rab37 vesicles for cancer immunotherapy
Source: Theranostics. 2022 Jan 1;12(1):340–61. doi: 10.7150/thno.65522 (PMC8690922; doi:10.7150/thno.65522)
Supplement: Supplementary file 1 — Supplementary figures and tables. [file thnov12p0340s1.pdf]

Targeting protumor factor chitinase-3-like-1 secreted by Rab37 vesicles for cancer immunotherapy

Pei-Shan Yang, Min-Hua Yu, Ya-Chin Hou, Chih-Peng Chang, Shao-Chieh Lin, I-Ying Kuo, Pei-Chia Su, Hung-Chi Cheng, Wu-Chou Su, Yan-Shen Shan, Yi-Ching Wang

Supplemental Data information

Figure S1 Cytokine/chemokine array and cytometric bead array detect the secretion cargo candidates and inflammation program in *Rab37* WT and KO mice.

Figure S2 Evaluation of the purity and sensitivity of nCHI3L1 Abs by SDS-PAGE and indirect ELISA.

Figure S3 Rab37 mediates CHI3L1 trafficking in macrophages in a GTPase-dependent manner.

Figure S4 nCHI3L1 Ab enhances $CD8^+$ T cell activity, inhibits expression of M2 marker gene in BMDMs and inactivates AKT in various cancer cell lines, but does not alter the viability of splenocytes and BMDMs.

Figure S5 nCHI3L1 Ab reduces angiogenesis of endothelial cells comparable to bevacizumab *in vitro*.

Figure S6 nCHI3L1 Ab suppresses tumor growth comparable with anti-CTLA-4 antibody in subcutaneous mouse tumor model.

Figure S7 nCHI3L1 Ab modulates tumor infiltrating macrophages *in vivo*.

Table S1 The plasmids and their characteristics used in the current study.

Table S2 Antibodies and their reaction conditions used in the current study.

Table S3 Clinicopathological parameters in lung, pancreatic and colon cancer patients recruited in the current study.

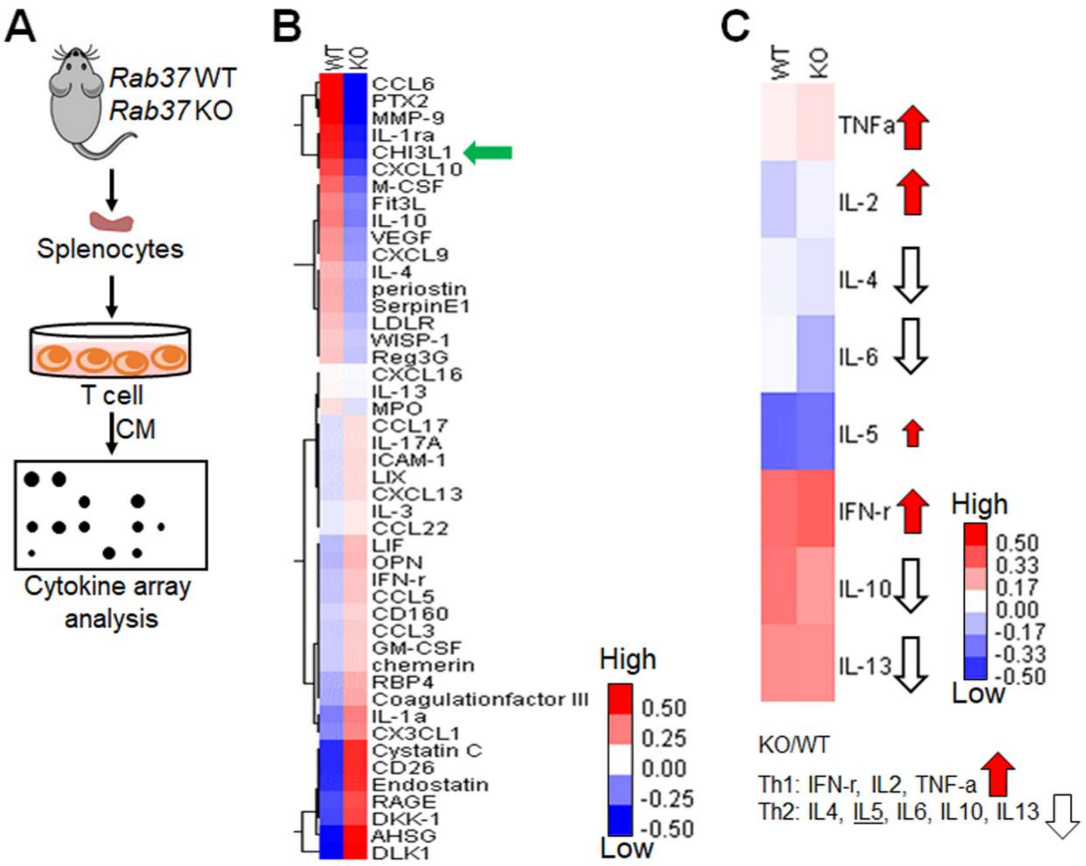

Figure S1. Cytokine/chemokine array and cytometric bead array detect the secretion cargo candidates and inflammation program in *Rab37* WT and KO mice. (**A, B**) The scheme (A) and heatmap (B) showing the secretin level of cytokines/proteins in CM derived from splenocytes from *Rab37* WT and KO mice. CHI3L1 is indicated by an arrow. (**C**) FLOW assay of Th1/Th2 markers in PBMC derived from *Rab37* WT and KO mice. *Rab37* KO mice correlated with Th1 inflammation program.

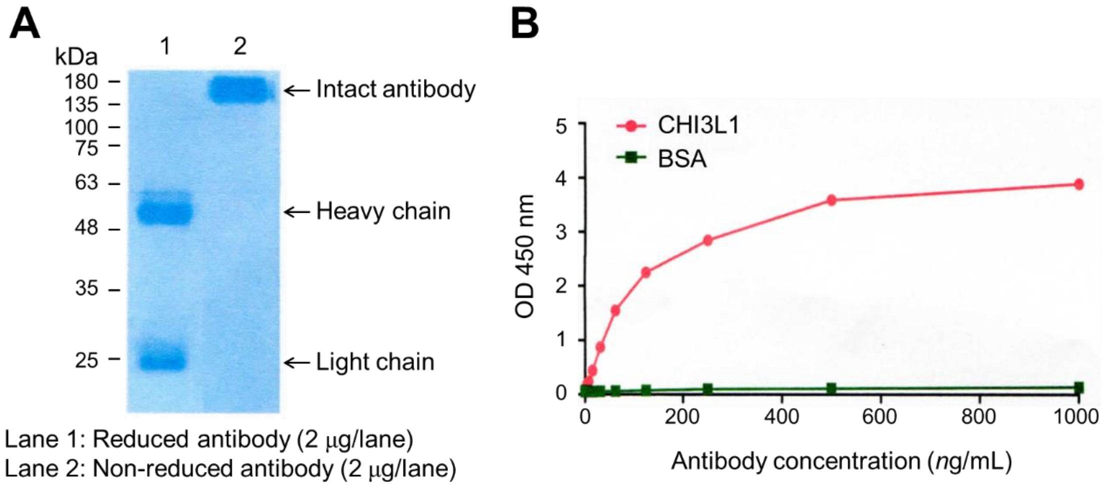

Figure S2. Evaluation of the purity and sensitivity of nCHI3L1 Abs by SDS-PAGE and indirect ELISA. (**A**) The purified nCHI3L1 Abs were subjected to SDS-PAGE analysis under the reduced (lane 1) and non-reduced (lane 2) conditions. Arrows indicate the predicted molecular weight of intact nCHI3L1 Ab, heavy chain and light chain. (**B**) The binding specificity of these antibodies to CHI3L1 was determined by indirect ELISA. Coating antigens were CHI3L1 and BSA. The curve of binding affinity is shown for different dilutions of nCHI3L1 Abs.

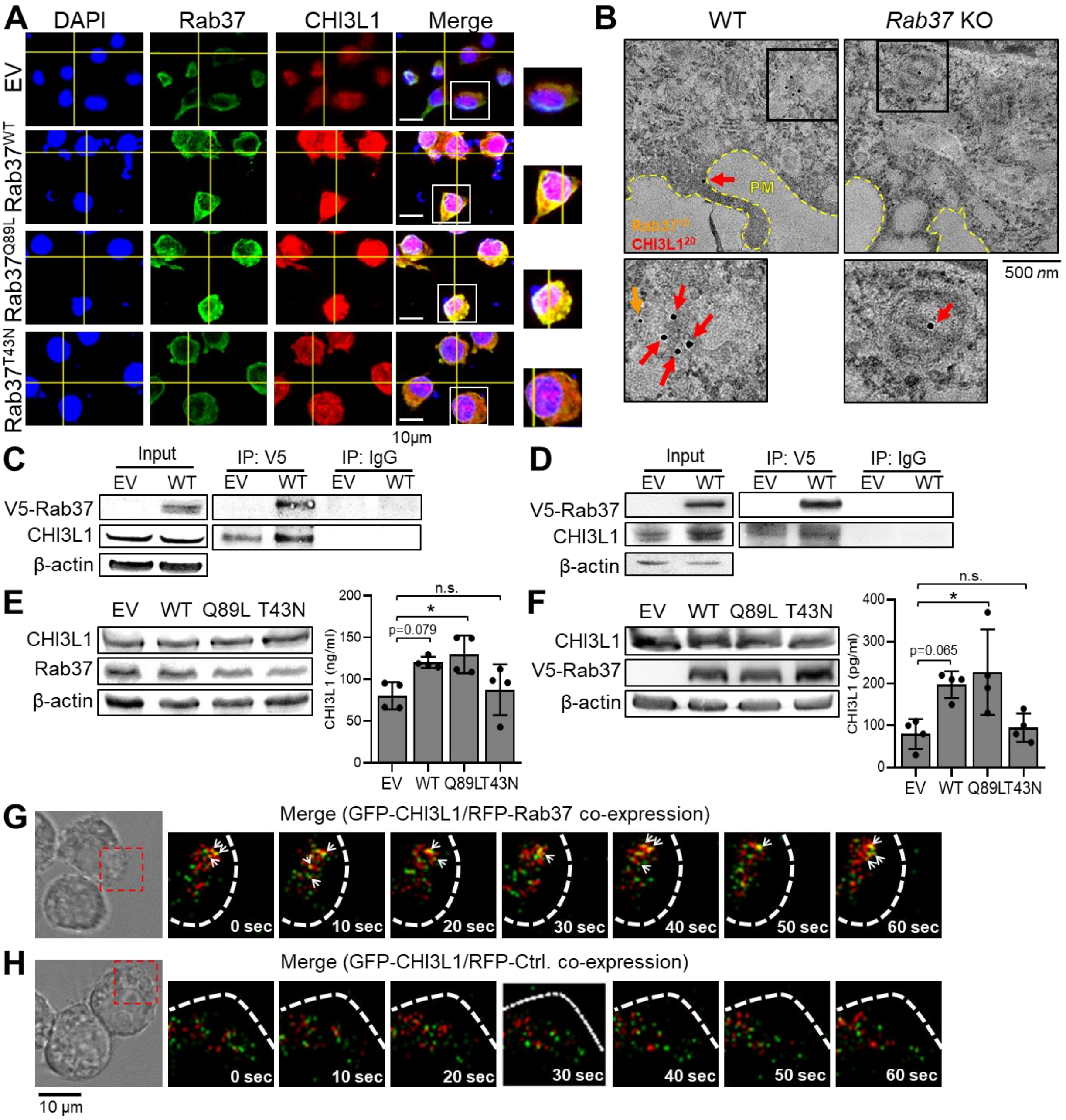

Figure S3. Rab37 mediates CHI3L1 trafficking in macrophages in a GTPase-dependent manner. (**A**) Confocal microscopy images of Rab37 (green), CHI3L1 (red) and nucleus staining (blue) in EV, Rab37^{WT}, Rab37^{Q89L} or Rab37^{T43N} RAW264.7 macrophage cells. Enlarged images of merged panel are shown. Scale bars: 10 µm. (**B**) Ultrastructural localization of Rab37 (10 nm of gold, yellow arrow) and CHI3L1 (20 nm of gold, red arrow) illustrated by immuno-EM images of control *Rab37* WT (*Left*) or KO (*Right*) BMDMs (H). PM: plasma membrane. Scale bars: 500 *n*m. Enlarged images of insets are shown. (**C, D**) Vesicles of EV or Rab37^{WT} THP1 (C) and

RAW264.7 cells (D) expressing V5-tagged Rab37 were collected by centrifugations and immunoprecipitated (IP) with anti-V5 and vesicle lysates were blotted for V5-Rab37 and endogenous CHI3L1. (**E, F**) CM-ELISA from EV, $Rab37^{WT}$, $Rab37^{Q89L}$ or $Rab37^{T43N}$ THP1 (E) and RAW264.7 cells (F) were performed to validate the level of CHI3L1 in CM. Immunoblots are shown for the cytosolic level of Rab37 and CHI3L1 protein (*Right*). (**G, H**) Selected frames from time-lapse confocal movies of RAW264.7 cells co-transfected with GFP-tagged CHI3L1 and RFP-tagged $Rab37^{WT}$ (G) or EV (H). Enlarged images of the boxed areas with time intervals in seconds are shown. Arrow indicates trafficking vesicle. Scale bars: 10 μm. Data represent mean ± SD. Data represent mean ± SD. * $p < 0.05$; n.s. non-significance, one-way ANOVA test.

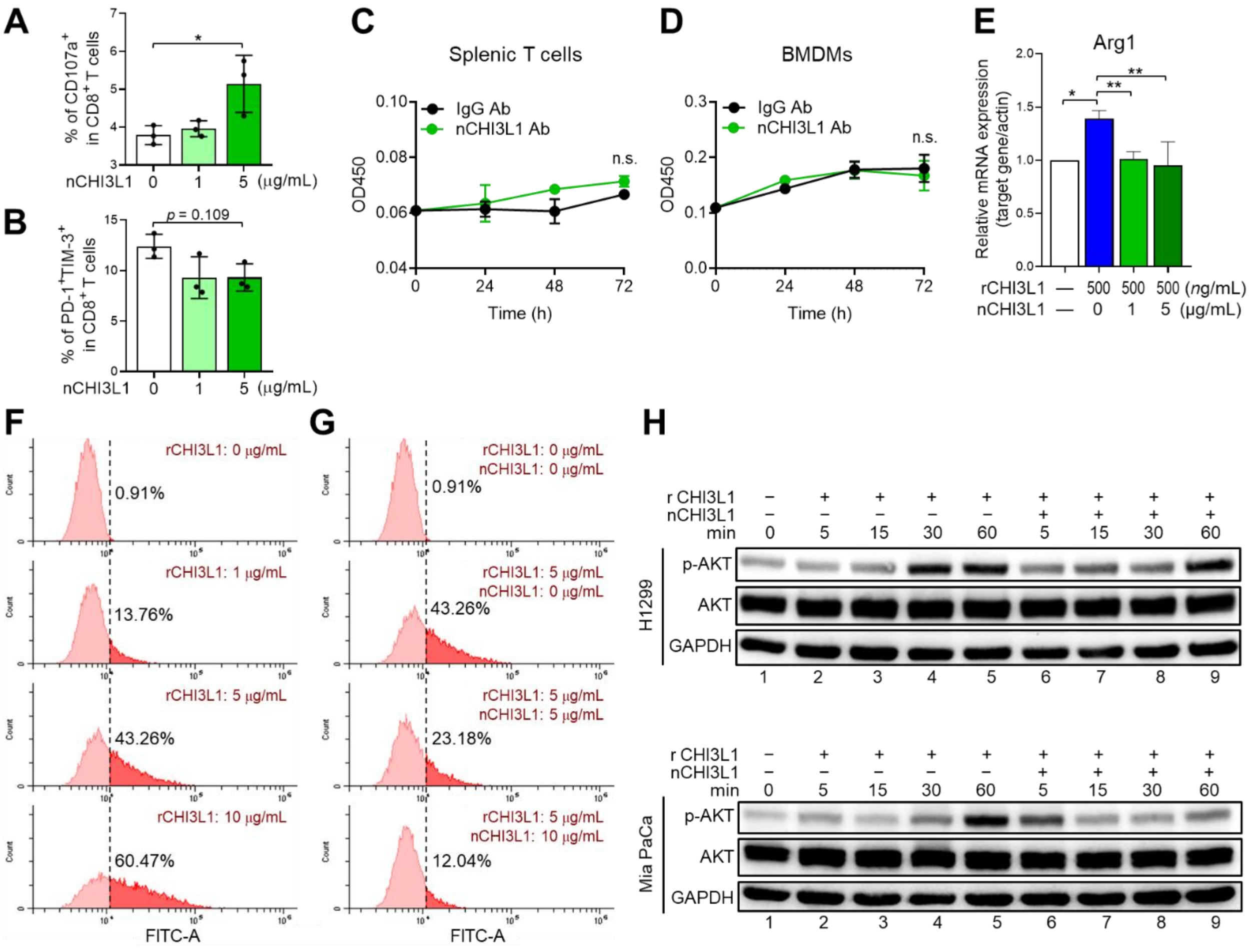

Figure S4. nCHI3L1 Ab enhances CD8$^+$ T cell activity, inhibits expression of M2 marker gene in BMDMs and inactivates AKT in various cancer cell lines, but does not alter the viability of splenocytes and BMDMs. (**A, B**) CD107a$^+$ functional CD8$^+$ cells increased (A) and PD-1$^+$Tim-3$^+$ exhausted CD8$^+$ cells decreased (B) upon treatment with CHI3L1 neutralizing antibody (nCHI3L1 Ab) for 24 h. (**C, D**) Cell viability measured by cell counting kit 8 (CCK-8) of splenocytes (C) and BMDM (D). (**E**) RT-qPCR assay showing nCHI3L1 Ab reduced the expression of M2 marker gene *Arginase-1* (*Arg1*) in BMDMs. (**F, G**) FACS analyses of the binding of recombinant CHI3L1 protein (rCHI3L1) with the cell surface receptor in H1299 lung cancer cells. Median florescence intensity was quantified and normalized to show rCHI3L1 binding (F) and inhibition by CHI3L1 neutralizing antibody (nCHI3L1) (G). (**H**) nCHI3L1 (1 μg/mL) inhibited AKT phosphorylation induced by rCHI3L1 (100 *n*g/mL) during the treatment period as indicated in H1299 lung cancer and MiaPaCa pancreatic cancer cells. Data represent mean ± SD. * $p < 0.05$; ** $p < 0.01$; *** $p < 0.001$; n.s. non-significance, one-way ANOVA test.

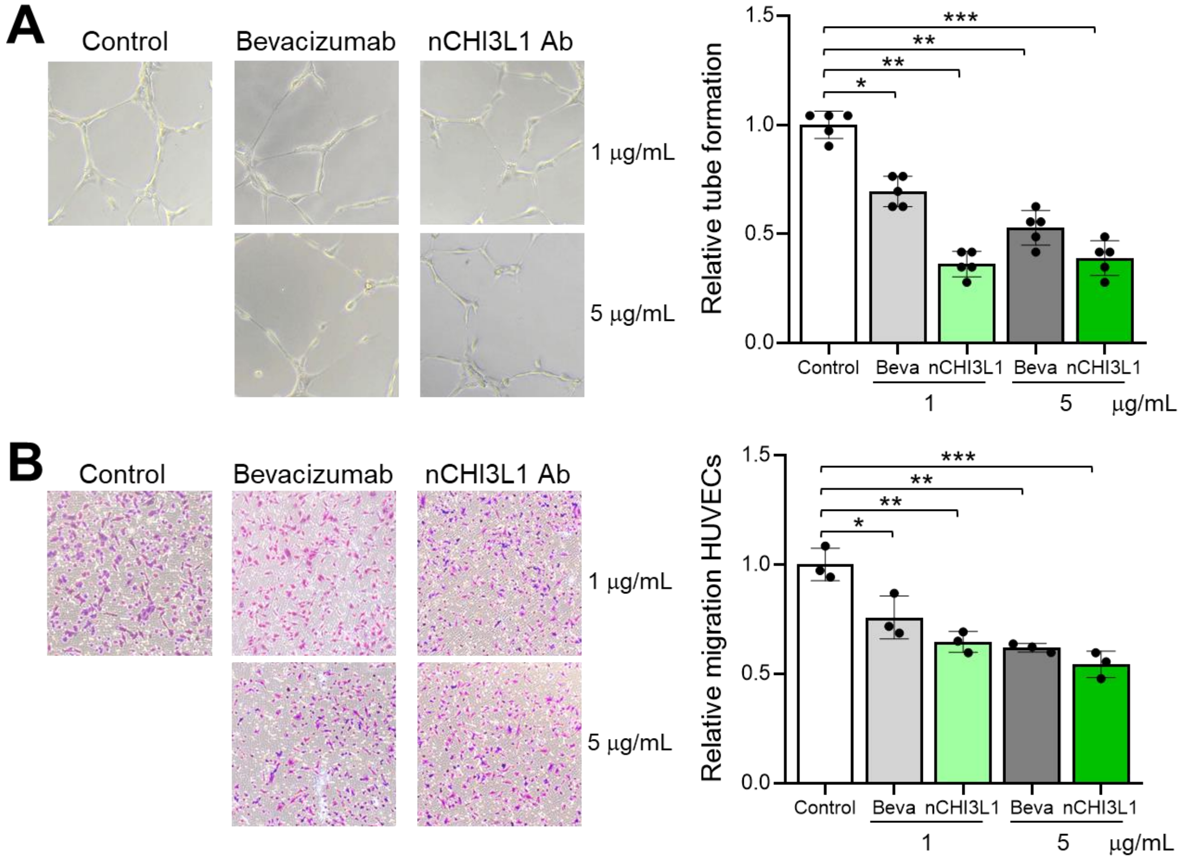

Figure S5. nCHI3L1 Ab reduces angiogenesis of endothelial cells comparable to bevacizumab *in vitro*. (**A**) Treatment with nCHI3L1 Abs reduced tube formation ability of human umbilical vein endothelial cells (HUVECs) in a dose-dependent manner compared with control group. Treatment with bevacizumab served as positive control. The tube formation was monitored at 6 h with each group quantified for the tube density/field from five independent experiments (*Right*). (**B**) Transwell migration assay of HUVECs (upper chamber) were cultured with cancer cells (lower chamber) which served as chemoattractants. Incubation of nCHI3L1 Ab reduced HUVEC migration in a dose-dependent manner. Bevacizumab was included for comparison. Migration ability was monitored at 16 h with each group quantified by comparison with initial seeding number of HUVECs (right). Data represent mean ± SD. * $p < 0.05$; ** $p < 0.01$; *** $p < 0.001$, one-way ANOVA test.

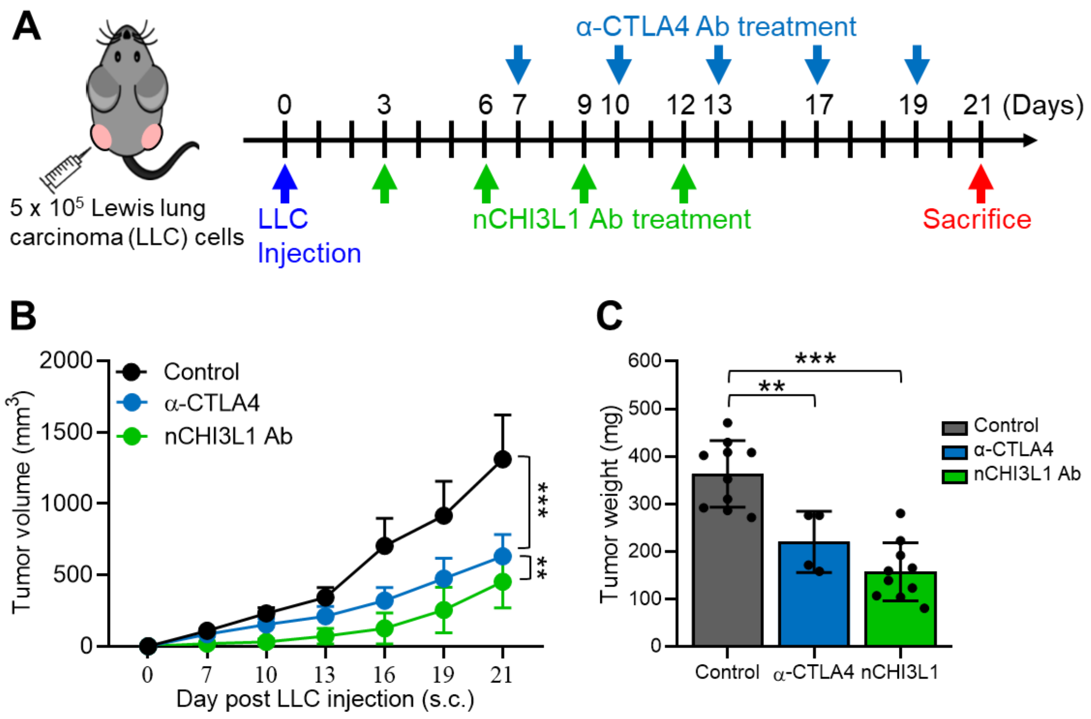

Figure S6. nCHI3L1 Ab suppresses tumor growth comparable with anti-CTLA-4 antibody in subcutaneous mouse tumor model. (A) LLC (5 x 10^5) subcutaneous model in *C57BL/6* mice with nCHI3L1 Ab or anti-CTLA-4 antibody (α-CTLA-4) treatment with the administration protocol as indicated in the scheme. **(B, C)** Tumor volume (B) and tumor weight (C) of LLC allograft in *C57BL/6* mice upon IgG (black), α-CTLA-4 (blue) or nCHI3L1 Ab (green) treatment. Data represent mean ± SD. ** $p < 0.01$; *** $p < 0.001$, two-way ANOVA test (B); one-way ANOVA test (C).

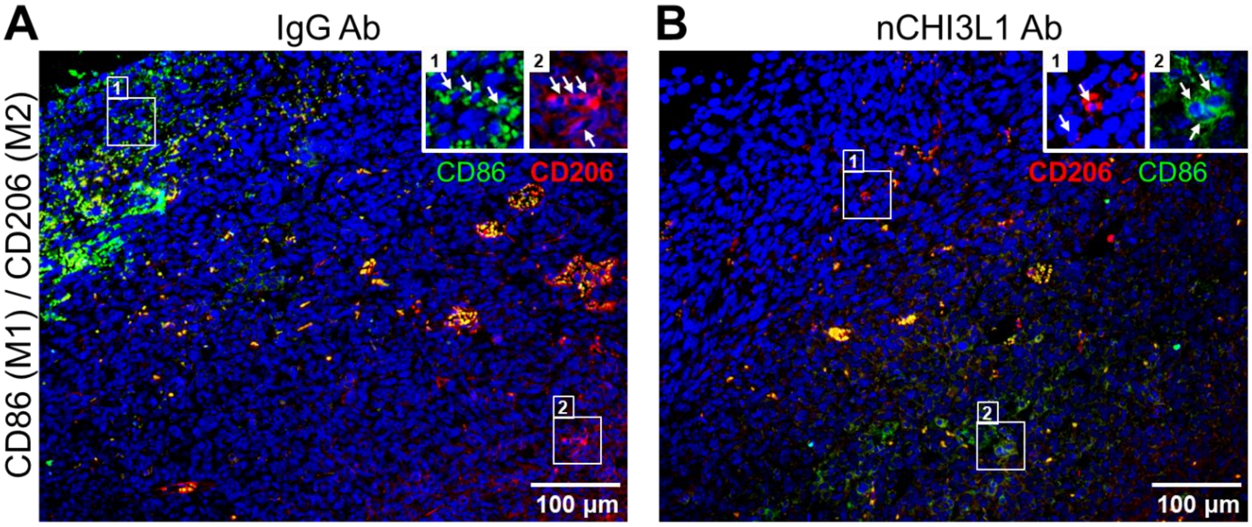

Figure S7. nCHI3L1 Ab modulates tumor infiltrating macrophages *in vivo*. (**A, B**) Distribution of CD86$^+$ M1 (green) and CD206$^+$ M2 (red) macrophages in IgG control group (A) and nCHI3L1 Ab treatment group (B). Enlarged images shown in insets of the representative regions are numbered as indicated. Blue fluorescence represents the nuclear staining. Scale bars: 100 μm.

Table S1. The plasmids and their characteristics used in the current study.

Plasmid	Target	Insert (bp)	Function	Source
pcDNA3.1-V5/His-vector	None	-- [A]	Vector control	Invitrogen
pcDNA3.1-V5/His-hRab37^{WT}	Human Rab37^{WT}	672	Overexpression	This work [B]
pcDNA3.1-V5/His-hRab37^{Q89L}	Human Rab37^{Q89L}	672	Overexpression	This work [B]
pcDNA3.1-V5/His-hRab37^{T43N}	Human Rab37^{T43N}	672	Overexpression	This work [B]
pcDNA3.1-V5/His-mRab37^{WT}	Mouse Rab37^{WT}	672	Overexpression	This work [B]
pcDNA3.1-V5/His-mRab37^{Q89L}	Mouse Rab37^{Q89L}	672	Overexpression	This work [B]
pcDNA3.1-V5/His-mRab37^{T43N}	Mouse Rab37^{T43N}	672	Overexpression	This work [B]
pCMV3-C-hCHI3L1-HA	Wild type human CHI3L1	1152	Overexpression	Sino Biological Inc.
pCMV3-C-mCHI3L1-HA	Wild type mouse CHI3L1	1170	Overexpression	Sino Biological Inc.
pDsRed2-C1-vector	None	-- [A]	Vector control	NovoPro Bioscience Inc.
pDsRed2-C1-hRab37^{WT}	Human Rab37^{WT}	672	TIRF	This work [C]
pDsRed2-C1-mRab37^{WT}	Mouse Rab37^{WT}	672	Real-time confocal	This work [C]
pCMV3-hCHI3L1-GFPSpark	Wild type human CHI3L1	1152	TIRF	Sino Biological Inc.
pCMV3-mCHI3L1-GFPSpark	Wild type mouse CHI3L1	1170	Real-time confocal	Sino Biological Inc.

[A] The plasmid is used as a backbone vector therefore there is no inserted DNA fragment.
[B] Rab37-WT, Q89L or T43N was PCR-amplified with designated mutation at the primer sequences and cloned into pcDNA3.1-V5/His expression vector to generate V5/His-tagged Rab37 expression vector.
[C] Rab37 cDNA was PCR-amplified and cloned into pDsRed2-C1 expression vector to generate RFP-tagged Rab37 expression vector.

Table S2. Antibodies and their reaction conditions used in the current study.

Target	KD	Raised in	Application	Dilution	Source	Catalog no.
Rab37	27	Rabbit	Western blot	1:1000	Proteintech	13051-1-AP
			Immunofluorescence	1:2000		
			Immunofluorescence-IHC	1:5000		
		Mouse	Immunofluorescence	1:2000	LCK BioLaboratories	mRab37
CHI3L1	24	Rabbit	Western blot	1:1000[b]	Abcam	ab77528
			Immunofluorescence	1:1000		
			Immunofluorescence-IHC	1:1000		
V5 tag	--[A]	Mouse	Western blot	1:5000	Invitrogen	46-0705
			Immunoprecipitation	1:150		
6X-His	--[A]	Mouse	Flow cytometry (FITC)	1:200	Abcam	ab1206
10 nm immune-Gold	--[A]	Mouse	Immuno-EM	1:30	Abcam	ab27241
20 nm immune-Gold	--[A]	Mouse	Immuno-EM	1:30	Abcam	ab27237
p-AKT	60	Rabbit	Western blot	1:2000	Cell signaling	4060s
AKT	60	Rabbit	Western blot	1:1000	Cell signaling	9272s
p-ERK	42	Rabbit	Western blot	1:1000	Cell signaling	9101s
ERK	42	Rabbit	Western blot	1:1000	Upstate	06-182
β-catenin	--[A]	Rabbit	Immunofluorescence	1:2000	Abcam	ab76315
NF-κB	--[A]	Rabbit	Immunofluorescence	1:5000	Cell Signaling	8242
CD4	--[A]	Rat	Flow cytometry (FITC)	1:200	BD Bioscience	553047
	--[A]	Rabbit	Immunofluorescence-IHC	1:3000	Abcam	ab183685

CD8	-- [A]	Rat	Flow cytometry (FITC)	1:200	BD Bioscience	553030
		Rabbit	Immunofluorescence-IHC	1:3000	Abcam	ab217344
CD107a	-- [A]	Mouse	Flow cytometry (PE)	1:200	BioLegend	121626
CD11b	-- [A]	Rat	Flow cytometry (BB515)	1:200	BD Bioscience	564454
CD25	-- [A]	Rat	Flow cytometry (APC)	1:200	BD Bioscience	557192
CD86	-- [A]	Rat	Flow cytometry (BB700)	1:200	BD Bioscience	742120
		Mouse	Immunofluorescence-IHC	1:1000	Genetex	GTX34569
CD163	-- [A]	Mouse	Immunofluorescence-IHC	1:100	Leica	NCL-L-CD163
CD206	-- [A]	Rat	Flow cytometry (Alexa647)	1:200	BD Bioscience	565250
		Rabbit	Immunofluorescence-IHC	1:1000	Abcam	ab64693
PD-1	-- [A]	Mouse	Flow cytometry (APC)	1:200	BioLegend	135210
TIMP-3	-- [A]	Mouse	Flow cytometry (BV421)	1:200	BD Bioscience	747626
CTLA4	-- [A]	Hamster	Flow cytometry (PE)	1:200	BD Bioscience	561718
F4/80	-- [A]	Mouse	Immunofluorescence-IHC	1:3000	Genetex	GTX26640
Foxp3	-- [A]	Rat	Flow cytometry (PE)	1:200	BD Bioscience	560408
DAPI	-- [A]	-- [B]	Immunofluorescence	-- [B]	Genetex	GTX30920
CD31	-- [A]	Human	DAB-IHC [C]	1:200	Abcam	ab28364
β-actin	42	Mouse	Western blot	1:5000	Genetex	GTX26276
GAPDH	37	Mouse	Western blot	1:1000	Santa Cruz	Sc-32233

[A] Molecular weight is not applicable to this antibody in such an application.

[B] DAPI is a commercial product for nuclear staining.

[C] DAB-IHC: 3,3’-diaminobenzidine tetrahydrochloride was the chromagen used.

Table S3. Clinicopathological parameters in lung, pancreatic and colon cancer patients recruited in the current study.

Clinical Parameters \ Patient number		Lung cancer	Pancreatic cancer	Colon cancer
		**161**	**155**	**180**
**Age**	< 65	91	74	78
	≥ 65	70	81	102
**Sex**	Male	76	85	104
	Female	85	70	76
**Tumor stage**	I-II	95	124	89
	III-IV	66	31	91
**T stage**	I-II	136	-[A]	49
	III-IV	25	-[A]	131
**N stage**	N0	83	-[A]	96
	≥ N1	78	-[A]	84
**M stage**	M0	150	58	133
	≥ M1	11	97	47
**Recurrence**	No	38	86	134
	Yes	123	69	46

[A] Clinical information was not available.
